# Astaxanthin Alleviates Lead‐Induced Toxicity by Restoring Hepatic and Gut–Liver Axis Homeostasis Through Multidimensional Metabolic and Antioxidative Pathways

**DOI:** 10.1002/fsn3.70971

**Published:** 2025-09-26

**Authors:** Zhongyang Du, Yan Sun, Xiaoli Zhu, Mengjing Liang, Daming Shi, Chunhui Zhang, Chunli Ji, Hongli Cui, Jinai Xue, Runzhi Li, Xiaoyun Jia

**Affiliations:** ^1^ College of Agriculture, Shanxi Agricultural University Shanxi Engineering Research Center for Genetics and Metabolism of Special Crops Taigu Shanxi China; ^2^ College of Food and Bioengineering, Yantai Institute of Technology Yantai Shandong China; ^3^ Key Laboratory of Coastal Biology and Biological Resource Utilization, Yantai Institute of Coastal Zone Research Chinese Academy of Sciences Yantai Shandong China

**Keywords:** antioxidation, astaxanthin, gut microbiota, hepatoprotection, lead poisoning, metabolomics, transcriptomics

## Abstract

Lead (Pb) poisoning is a major public health concern of environmental origin in the world. It is essential to develop effective ways such as utilizing natural products as therapeutic agents for prevention and therapy of Pb‐induced diseases. This study explores the effects and underlying mechanisms of astaxanthin (ATX), a natural compound with potent antioxidant properties, in alleviating Pb‐induced toxicity in model mice. Supplementation with ATX significantly ameliorated lead‐induced physiological and biochemical disruptions, including weight loss, hepatic and renal damage, and metabolic imbalances. Metabolomic and transcriptomic analyses revealed that ATX played a positive role in improving redox homeostasis, regulating lipid, amino acid, and nucleotide metabolism, and activating critical pathways such as Nrf2/ARE, PPAR, and S1P, thereby enhancing the antioxidative, anti‐inflammatory, and detoxification capacities of the mice. ATX supplementation also modulated mouse gut microbiota by promoting beneficial bacterial populations, suppressing harmful strains, and increasing short‐chain fatty acid production, thereby effectively restoring gut‐liver axis balance. These findings demonstrate that ATX possesses comprehensive activities against lead toxicity via multi‐dimensional regulatory mechanisms, highlighting ATX as a promising therapeutic agent for heavy metal poisoning. Further research is warranted to validate the clinical applications of ATX and evaluate its long‐term safety.

## Graphical abstract

This study firstly reveals astaxanthin roles in alleviating lead toxicity in mouse via multiple approaches. Astaxanthin significantly mitigates metabolic and inflammatory imbalances through gut‐liver axis modulation. Astaxanthin activates Nrf2/ARE and PPAR pathways, providing multidimensional antioxidative and metabolic protection. Astaxanthin improves gut microbiota functions, promoting beneficial bacteria to reduce systemic inflammation. Astaxanthin has potential as a safe and effective agent for heavy metal toxicity therapy.

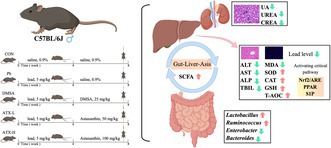

## 1 Introduction

Lead (Pb), a highly toxic heavy metal, is a global health concern of environmental origin due to its non‐degradability and bioaccumulation in organisms (Kumar et al. 2020; Pan et al. 2024). Vulnerable groups including children, pregnant women, and individuals with occupational exposure face heightened lead exposure risks. Pb can enter the body via inhalation, ingestion, or dermal contact. Pb accumulates in organs and causes multi‐system damage, with oxidative stress identified as a key pathogenic pathway (Fan et al. 2020; Nemsadze et al. 2009). Lead exposure disrupts antioxidant defenses by inhibiting key enzymes such as SOD, CAT, and GPx, resulting in ROS accumulation, which triggers lipid peroxidation, protein oxidation, and DNA damage, ultimately leading to cell death (Deza‐Ponzio et al. 2023; Wu et al. 2006). Additionally, lead activates inflammatory pathways, promoting the release of pro‐inflammatory cytokines and aggravating tissue inflammation (Wang, Zheng, et al. 2021; Simões et al. 2015). Lead also disrupts energy, lipid, and amino acid metabolism, leading to toxic substance buildup and impaired detoxification (Chen et al. 2014). Beyond these direct cellular effects, lead exposure disrupts the gut microbiota by increasing harmful bacteria, reducing beneficial bacterial populations, and compromising gut barrier function (Wang, Gao, et al. 2024). This gut dysbiosis impacts nutrient absorption and immune responses through the gut–liver axis, exacerbating liver and kidney damage (Ding et al. 2022). Current treatments for lead poisoning primarily involve chelating agents like calcium disodium EDTA and meso‐2,3‐dimercaptosuccinic acid (DMSA), which aid in lead excretion (Bradberry and Vale 2009). However, chelation therapy has several limitations. For example, chelation therapy cannot reverse lead‐induced tissue damage and may also cause depletion of essential metal ions (e.g., zinc and iron). Furthermore, prolonged use of such treatment may lead to nephrotoxicity and hepatotoxicity (Chisolm Jr. 1990). Recently, the use of natural products as therapeutic agents is rapidly growing, showing a promising application in healthcare. Consequently, there is an increasing need to identify natural bioactive compounds as safe and effective agents to protect organ function and mitigate the toxic effects of lead.

Astaxanthin (ATX), a natural carotenoid found in marine organisms such as algae, yeast, salmon, shrimp, and crab (Ambati et al. 2014), offers potential as an adjunct therapy for lead poisoning due to its super antioxidant activity, which is several hundred times stronger than that of vitamin E. ATX can effectively scavenge free radicals and protect cell membranes and DNA from oxidative damage (Dose et al. 2016). Beyond antioxidant effects, ATX exhibits anti‐inflammatory, anti‐apoptotic, immunomodulatory, and metabolic regulatory properties (Li et al. 2015). Notably, ATX can inhibit inflammation pathways, such as NF‐κB and MAPK, reducing pro‐inflammatory cytokines and cell apoptosis (Chang and Xiong 2020). ATX also activates peroxisome proliferator‐activated receptor (PPAR) pathways to enhance lipid and glucose metabolism, supporting cellular energy balance (Le Goff et al. 2019). As a natural compound, ATX was found to prevent high‐fat diet‐induced hepatic steatosis, obesity, and muscle dysfunction in mice by modulating lipid metabolism and gut microbiota (Wang, Ma, et al. 2021, 2022; Chen et al. 2023). However, it remains unknown whether ATX can be used as an effective agent for the treatment of lead‐induced toxicity.

In this study, we established a lead‐exposed mouse model to systematically evaluate the protective effects and molecular mechanisms of ATX against lead‐induced multisystem damage. We conducted comprehensive physiological and biochemical assessments to quantify the impact of ATX on overall health, along with histological analyses to determine its protective effects on liver and kidney tissues. Using transcriptomic and metabolomic analyses, we investigated ATX‐mediated regulation of hepatic gene expression and metabolism in hepatic tissue. Moreover, 16S rRNA gene sequencing was performed to examine ATX's role in modulating the gut microbiota and its connection to the gut–liver axis. Through these multifaceted approaches, we aimed to elucidate the precise mechanisms by which ATX mitigates lead‐induced toxicity and improves health, thereby providing a robust theoretical foundation and potential therapeutic strategies for clinical treatment of lead‐induced diseases.

## 2 Materials and Methods

### 2.1 Chemicals and Reagents

ATX oleoresin was obtained from Shandong Jinjing Biotechnology Co. Ltd. (product name: Astalgae astaxanthin oleoresin). The purity of ATX = (831.90 + 13.10)/(831.90 + 47.40 + 13.10) was 94.69%; the ratio of trans‐ATX reached 93.22%, while the ratio of 9‐cis‐ATX was only 1.47%. The high purity of ATX met the requirements of further study. Serum total triglycerides (TG), total cholesterol (TC), alkaline phosphatase (ALP), alanine aminotransferase (ALT), aspartate aminotransferase (AST), total bilirubin (TBIL), uric acid (UA), creatinine (CREA), urea (UREA), liver tissue malondialdehyde (MDA), total antioxidant capacity (T‐AOC), reduced glutathione (GSH), superoxide dismutase (SOD), and catalase (CAT) were purchased from Nanjing Jiancheng Bio‐Engineering Institute Co. Ltd. (Nanjing, China). Trizol reagent was obtained from Shenggong BBI Life; a PrimeScript RT Reagent kit with gDNA Eraser was purchased from Takara Biomedical Technology (Beijing) Co. Ltd. Hematoxylin and eosin (H&E) and Oil‐red O were obtained from Hongci Medical Service Co. Ltd. (Anhui Chaohu, China) Other chemicals were analytical grade unless otherwise specified and were obtained from commercial sources.

### 2.2 Animals and Experimental Design

Forty male C57BL/6 mice (20 ± 2 g, specific pathogen‐free) were obtained from Shanxi Medical University. Mice were housed under controlled conditions (22°C ± 2°C, 50% ± 5% humidity, 12 h light/dark cycle) at Shanxi Agricultural University, with free access to food and water. All procedures were approved by the Institutional Animal Care and Use Committee (Approval Number: SXAU‐EAW‐2021‐116). After 1 week of acclimatization, mice were randomly divided into a normal group (*n* = 8) and a lead‐exposed group for 4 weeks. Successful modeling was confirmed by measuring lead content in urine and blood. Experimental diets were obtained from Beijing Huafukang Bioscience Co. Ltd., and their composition is provided in Table S1. This experiment was designed using the body surface area (BSA)‐based dose conversion principle (conversion factor *K* = 7.0) (Reagan‐Shaw et al. 2008; Harloff‐Helleberg et al. 2017), integrated with the safety assessment framework for bioactive substances established by Brendler and Williamson (Brendler and Williamson 2019), to optimize the astaxanthin administration concentration. In conjunction with the literature‐reported effective dose range of 25 to 100 mg/kg body weight (BW) (Wu et al. 2020), the final experimental intervention doses were determined as 25 and 50 mg/kg. Toxicological evaluation data demonstrate that these dose levels are significantly below the no‐observed‐adverse‐effect level (NOAEL = 100 mg/kg/day) for rodents (Brendler and Williamson 2019), and after cross‐species dose correction, they remain within the safety threshold of the human acceptable daily intake (ADI). Post‐modeling, mice were assigned to five groups as follows: CON [Non‐Pb plus intragastric saline (50 mg/kg BW)], Pb [Pb exposure plus intragastric saline (50 mg/kg BW)], DMSA [Pb exposure plus dimercaptosuccinic acid (25 mg/kg BW)], ATX‐L [Pb exposure plus astaxanthin (25 mg/kg BW)] and ATX‐H [Pb exposure plus astaxanthin (50 mg/kg BW)]. All treatments were administered daily via intragastric gavage (200 μL/mouse). Mice were fasted for 12 h before humane euthanasia under isoflurane anesthesia. The experimental protocol is illustrated in Figure S1.

### 2.3 Sample Collection and Determination of Pb Content

Lead content was measured at two key points: after lead exposure modeling and at the end of the experiment. For model confirmation: urine samples were collected using the reflex urination method and stored in sterile tubes. Blood samples were obtained from the posterior orbital venous plexus under local anesthesia with 1% procaine hydrochloride. Blood was centrifuged (4000 rpm, 10 min, 4°C) to separate serum for lead measurement. For final sample collection: after a 12‐h fast, mice were euthanized under isoflurane anesthesia. Whole blood was collected via the abdominal aorta using tubes with and without anticoagulant. Plasma (in sodium heparin tubes) and serum (clotted at room temperature) were separated by centrifugation (4000 rpm, 10 min, 4°C) and stored at −80°C for analysis. The lead content in whole blood, liver, and urine was analyzed using an atomic absorption spectrophotometer (AA‐6300; Shimadzu, Japan), based on the established methods (Hassona and El‐Wahed 2023).

### 2.4 Measurement of Body Weight and Food Intake, Sampling of Blood and Organs/Tissues

The body weight of each mouse was recorded every 2 days after an overnight fast. To ensure data reliability, the measurements were independently performed in triplicate. Each group was provided with a total of 30 g of diet daily, and the remaining food was measured the following day to assess consumption.

At the end of the experiment, all mice were sacrificed following isoflurane anesthesia. Once the blink reflex disappeared and no response was observed to hind limb clamping using hemostatic forceps, the abdomen was incised with tissue scissors to expose the abdominal aorta. Whole blood was then collected using two types of vacuum blood collection tubes: (1) Tubes containing sodium heparin: Blood was centrifuged at 4000 rpm for 10 min at 4°C to separate plasma, which was stored at −80°C for further analysis of coagulation and electrolyte markers. (2) Tubes without anticoagulant: Blood was allowed to clot at room temperature, followed by centrifugation at 4000 rpm for 10 min at 4°C to obtain serum, which was stored at −80°C for liver and kidney function assessments.

After sacrifice, all samples were dissected on a sterile workbench (Sujing, China). The peritoneum was opened, and the liver was carefully isolated, washed with cold saline (pH 6.9), and divided into four equal sections. One section was allocated for histopathological analysis and TUNEL assay, while the remaining sections were reserved for antioxidant activity assays, mRNA expression analysis, and transcriptomic and metabolomic studies. Additionally, the liver, heart, kidneys, and spleen were individually collected and weighed, then rapidly frozen in liquid nitrogen and stored at −80°C for future analysis.

### 2.5 Hematological Analysis

The hemocytometer and cyanmethemoglobin protocols were followed to estimate the red blood cell (RBC) count, white blood cell (WBC) count, and hemoglobin (HGB) concentration (Thrall and Weiser 2002). The red blood cell indices, i.e., the mean corpuscular volume (MCV), the mean corpuscular hemoglobin (MCH), and the mean corpuscular hemoglobin concentration (MCHC) were then computed. Additionally, differential leukocyte count was done manually by blood smears stained with Giemsa staining (Feldman et al. 2000).

### 2.6 Assessment of Kidney Histopathological Examination

Kidney samples were immersed in 4% paraformaldehyde for 24 h, dehydrated using gradient ethanol, cleared with xylene, and embedded in paraffin wax blocks. Five‐micron‐thick sections were prepared using a microtome (Leica, Germany). After dewaxing in xylene and rehydrating through decreasing grades of ethanol, the sections were stained with H&E and then subjected to gradient alcohol and xylene dehydration. The samples were observed, and the images were captured using a light microscope (Leica, Germany).

### 2.7 Assessment of Liver Oxidation Resistance and Liver Histopathological Examination

Some liver samples were ground at 4°C, and the supernatant was collected after centrifugation (3500 rpm for 10 min) and T‐AOC, SOD, CAT, GPX, and MDA levels were determined using corresponding kits (Jiancheng, China) to determine the oxidation resistance index in the liver. The processing of liver samples for liver histopathology was identical to that used for kidney samples. Liver and kidney tissue damages were assessed using a blinded evaluation method and scored in all tissue sections using 5 random non‐overlapping fields from each slide at ×200 and ×400 magnifications, respectively. A lesion scoring system was performed for the liver and kidney tissue damage assessment (Hur et al. 2013; Abo‐El‐Sooud et al. 2023).

### 2.8 Evaluation of Hepatocellular Apoptosis

The five‐micron‐thick sections of the liver were used as materials. Hepatocellular apoptosis was evaluated using the YF488 TUNEL assay apoptosis detection kit. After the TUNEL reaction, the samples were stained with 4′,6‐di‐amidino‐2‐phenylindole (DAPI) and viewed under an inverted fluorescence microscope at excitation wavelengths of 358 and 485 nm. The cells were dyed with blue fluorescence at 358 nm, and the apoptotic cell was marked with green fluorescence at 485 nm. The images of each sample were captured during microscopy, and the cell number was counted by using the ImageJ software (v1.48; NIH, USA).

### 2.9 Liver Transcriptomic Analysis

Liver samples from the CON, Pb, DMSA, ATX‐L, and ATX‐H groups (*n* = 3) were processed as described by Chen et al. Chen et al. (2018), with RNA quality monitored on 1% agarose gels. Purity, concentration, and integrity of RNA were checked using a NanoPhotometer spectrophotometer (IMPLEN, CA, USA), Qubit RNA Assay Kit with Qubit 2.0 Fluorometer (Life Technologies, CA, USA), and Agilent 2100 Bioanalyzer system (Agilent Technologies, CA, USA), respectively. For library preparation, 1 μg of RNA per sample was processed with the NEBNext UltraTM RNA Library Prep Kit for Illumina(NEB, USA) following the manufacturer's protocol. Briefly, mRNA was isolated using poly‐T oligo magnetic beads, fragmented, and converted into cDNA. After adaptor ligation, fragments (250–300 bp) were selected, and PCR amplification was performed. Libraries were quality‐checked on the Bioanalyzer 2100 and clustered on a cBot System with TruSeq PE Cluster Kit. Sequencing was performed on an Illumina platform to generate 150 bp paired‐end reads. Analytical conditions followed methods by Yao et al. (2021) and Zhan et al. (2022).

### 2.10 Liver Metabolomic Analysis

Liver samples from the CON, Pb, DMSA, ATX‐L, and ATX‐H groups (*n* = 3) were processed following the previously described methods (Chen et al. 2013; Fraga et al. 2010). Samples stored at −80°C were thawed on ice, homogenized, and mixed with a methanol: water (7:3, v/v) solution containing an internal standard. After vortexing and centrifugation, the supernatant was collected, frozen at −80°C, and further centrifuged, with 200 μL prepared for LC–MS analysis. The samples were analyzed on an LC‐ESI‐MS/MS system (UPLC, ExionLC AD, https://sciex.com.cn/; MS, QTRAP System, https://sciex.com/) using a Waters ACQUITY UPLC HSS T3 C18 column at 40°C, a flow rate of 0.4 mL/min, and a binary solvent system of water and acetonitrile, both containing 0.1% formic acid in gradient elution. Mass spectrometry was conducted on a QTRAP LC–MS/MS with an ESI Turbo Ion‐Spray interface in positive and negative ion modes, using Analyst 1.6.3 software. Key parameters included a source temperature of 500°C, an ion spray voltage of 5500 V (positive) and −4500 V (negative), and gas pressures set at 55, 60, and 25 psi for GSI, GSII, and CUR, respectively. Tuning and calibration were performed with polypropylene glycol solutions, and specific multiple reaction monitoring (MRM) transitions were monitored for metabolites. The analytical conditions adhered to Yao et al. (2021) and Li et al. (2021).

### 2.11 Gut Microbiota Analysis

Gut contents were aseptically collected from the intestinal tract of sacrificed mice and sent to Shanghai Personal Biotech Co. Ltd. for 16S rRNA sequencing to assess microbial diversity. After DNA extraction, the concentration and purity of genomic DNA were measured, and bacterial 16S rDNA was amplified using universal primers on an Illumina MiSeq platform (2 × 300). Quality control was conducted on the FASTQ data, and fragments shorter than twice the read length were merged using FLASH software. Clustering was performed with QIIME1 and QIIME2, and sequences were clustered into operational taxonomic units (OTUs) at a 97% identity threshold using Uparse software. The observed species, Shannon and Simpson diversity indices, and weighted UniFrac principal coordinate analysis (PCA) were analyzed using QIIME (version 1.7.0) to evaluate microbiota structure. Linear discriminant analysis effect size (LEfSe) was used to identify significant differences between groups (Zhu et al. 2018), and representative sequences were aligned to compare OTU abundances. Data visualization was conducted using R software.

### 2.12 Determination of mRNA Expression Levels by qRT‐PCR

Total mRNA was extracted from liver tissue of the CON, Pb, DMSA, ATX‐L, and ATX‐H groups (*n* = 3) using Trizol reagent (Shenggong BBI Life, Shanghai, China) following the manufacturer's protocol. Approximately 0.1 g of liver tissue was ground into a fine powder using liquid nitrogen. The cDNA synthesis was performed using the PrimeScript RT Reagent Kit with gDNA Eraser (TaKaRa, Japan). Gene expression levels were quantified using the SYBR Premix Ex Taq II (TaKaRa, Japan) on a CFX96 Real‐Time PCR Detection System (Bio‐Rad, USA).

Primers were designed using Primer Premier 6.0 based on sequences retrieved from the NCBI GenBank database, with GAPDH as the reference gene. The primer sequences are shown in Table S2. qPCR reactions were conducted in 20 μL reaction volumes under standard cycling conditions, and relative gene expression levels were calculated using the $2^{-∆∆C_{t}}$ method.

### 2.13 Statistical Analysis

All experiments were biologically repeated three times, and the data were analyzed with SPSS Statistics 17.0 (IBM, Chicago, IL, USA) statistical software and are presented as the mean ± standard deviation (SD). Multiple comparisons among treatments were statistically analyzed by using Duncan's multiple range test in one‐way analysis of variance (ANOVA) (*p* < 0.05).

Transcripts with FDR < 0.05, *p* < 0.05 and fold change (FC) ≥ 2 were considered as differentially expressed genes (Benjamini‐Hochberg multiple testing correction). The DEseq2 algorithm was applied to filter the differentially expressed genes according to fragments per kilobase of transcript per million fragments mapped.

LC–MS data analysis was performed through Progenesis QI software (Waters, USA) for extracting, alignment, peak picking, and retention time adjustment. All metabolites were identified by comparison of “Score”, “Fragmentation Score”, “Mass Error (ppm),” and “Isotope Similarity” with the standard mass spectra available on the commercial databases mzCloud (https://www.mzcloud.org), HMDB (http://www.hmdb.ca), and KEGG (http://kegg.jp). The normalized data were analyzed with R language. Orthogonal partial least squares‐discriminant analysis (OPLS‐DA) was used to compare the metabolomic profiling among groups. Differential metabolites were then selected on the basis of VIP (variable importance in projection) ≥ 1 and FC ≥ 2 or FC ≤ 0.05. Graphs were generated using OriginPro 9.1 (OriginLab, USA).

## 3 Results

### 3.1 Effects of ATX Supplementation on Body Weight, Food Intake, and Lipid Levels, Lead Content in Blood, Urine, and Liver of the Pb‐Exposed Mice

Mouse body weight was monitored over 56 days (Figure S2A). During the first 4 weeks of lead exposure, no significant differences in body weight were observed among groups. However, from the fifth week, mice in the Pb and DMSA groups exhibited significantly lower body weights compared to the control group, and this trend persisted to the end of the experiment. ATX treatment mitigated weight loss, and the ATX‐L group maintained a weight similar to the control group by the experiment's end. Food intake remained unchanged across groups (Figure S2B).

Kidney weight in the DMSA group was lower than that in the Pb group (*p* < 0.05) (Table 1). ATX intervention increased liver and kidney weights by up to 17.86% and 11.54%, respectively, compared to the Pb group (*p* < 0.05), with no significant changes in other organs. These results suggest ATX mitigates weight loss and protects liver and kidney function without causing organ damage, consistent with the prior studies (Ellacott et al. 2010; Al‐Qahtani et al. 2022). After 4 weeks of lead exposure, blood and urine lead levels were significantly elevated in all lead‐exposed groups (Pb, DMSA, ATX‐L, ATX‐H) compared to the lead‐free control (Figure S2C), confirming successful model induction.

**TABLE 1:** Effect of ATX supplementation on relative organ weight and tissue damage parameters in mice with lead‐induced chronic lead poisoning^a^.

Group	CON	Pb	DMSA	ATX‐L	ATX‐H
*Relative organ weights*					
Heart (g)	0.16 ± 0.01^a^	0.15 ± 0.01^a^	0.16 ± 0.02^a^	0.16 ± 0.01^a^	0.16 ± 0.01^a^
Liver (g)	1.04 ± 0.07^a^	0.79 ± 0.03^b^	0.8 ± 0.05^b^	0.86 ± 0.07^b^	0.88 ± 0.1^b^
Spleen (g)	0.85 ± 0.06^a^	0.73 ± 0.06^a^	0.77 ± 0.06^a^	0.82 ± 0.08^a^	0.83 ± 0.06^a^
Kidney (g)	0.28 ± 0.02^a^	0.23 ± 0.02^b^	0.24 ± 0.01^ab^	0.28 ± 0.02^a^	0.26 ± 0.02^ab^
*Tissue damage score*					
Kidney tubular degeneration (TD) score	0.14 ± 0.04^d^	3.65 ± 0.07^a^	1.86 ± 0.24^b^	1.72 ± 0.18^b^	1.5 ± 0.16^c^
Kidney tubular necrosis (TN) score	0.11 ± 0.02^d^	3.68 ± 0.15^a^	1.95 ± 0.22^b^	1.72 ± 0.24^c^	1.57 ± 0.07^c^
Kidney tubulointerstitial inflammation (TIN) score	0.13 ± 0.01^e^	3.82 ± 0.1^a^	2 ± 0.25^b^	1.54 ± 0.19^c^	1.33 ± 0.1^d^
Kidney total histologic score (THS)	0.28 ± 0.06^e^	7.38 ± 0.12^a^	3.79 ± 0.45^b^	3.36 ± 0.27^c^	2.92 ± 0.28^d^
Liver tissue damage score	0.21 ± 0.06^d^	3.84 ± 0.07^a^	1.77 ± 0.28^b^	1.55 ± 0.15^c^	1.46 ± 0.15^c^

*Note:* The different letters (a, b) indicate significant differences (*p* < 0.05). Tubular degeneration (TD) and tubular necrosis (TN) score: Absence—score 0; Mild (0%–10%)—score 1; Moderate (10%–25%)—score 2; Severe (25%–50%)—score 3; Very severe (> 50%)—score 4. Tubulo‐interstitial inflammation (TIN) score: Absence—score 0; Mild (0%–5%)—score 1; Moderate (5%–10%)—score 2; Severe (15%–25%)—score 3; Very severe (> 50%) score 4. Total kidney histologic damage score (THS = TD/2 + TN + TIN/2): Normal—score 0–2; Mild—score 3–5; Moderate—score 6–8; Severe—9–10. Liver tissue damage score: Normal: score—0%; < 25% damage: score 1%; 25%–50% damage: score 2%; 50%–75% damage: score 3%; 75%–100% damage: score 4%.

The small different letters (a‐e) indicate the significant difference (p < 0.05) among the treatments.

^a^ All values are expressed as the means ± SDs (*n* = 6). Mean separation was performed using Duncan's multiple range test.

Serum analysis revealed lipid abnormalities in the Pb group, with a 39% reduction in total cholesterol (TC) and significantly lower triglyceride (TG) levels compared to the control (Figure S2D–F). ATX treatment improved TC and TG levels in a dose‐dependent manner, with the ATX‐H group showing similar levels to those in the control group (TC: ATX‐H = 3.775 mmol/L, control = 4.026 mmol/L; TG: ATX‐H = 1.452 mmol/L, control = 1.528 mmol/L). These results indicate that ATX effectively mitigates lead‐induced lipid disturbances (*p* < 0.05).

### 3.2 Effect of ATX Supplementation on Hematological Parameters in Pb‐Exposed Mice

A significant reduction (*p* < 0.05) in RBC counts (1.46‐fold lower) and HGB concentration (1.46‐fold lower), along with an increase in WBC counts (2.13‐fold higher) and lymphocytes (2.36‐fold higher), was detected in the Pb‐intoxicated group mice compared to the negative control group. ATX‐L and ATX‐H treatment normalized these parameters toward control levels (Table 2). The red blood cell indices (MCV and MCHC) were insignificantly different from the negative control group in all test groups (*p* < 0.05). However, differential leukocyte counts (neutrophils and monocytes) had the same trend compared to the control group and partially reversed the Pb‐induced increases.

**TABLE 2:** Effect of ATX supplementation on hematologic markers for lead‐induced chronic lead poisoning in mice^a^.

Group	WBC (10^9^/L)	Lymph (10^9^/L)	Mon (10^9^/L)	Gran (10^9^/L)	RBC (10^12^/L)	HGB (g/L)	MCV (fl)	MCHC (g/L)
CON	5.80 ± 1.47^c^	4.40 ± 0.46^c^	0.17 ± 0.06^c^	1.53 ± 0.15^a^	8.37 ± 0.34^a^	131.67 ± 6.11^a^	47.00 ± 2.30^a^	389.67 ± 10.79^a^
Pb	12.37 ± 0.51^a^	10.37 ± 0.67^a^	0.57 ± 0.12^a^	0.77 ± 0.06^c^	5.74 ± 0.48^c^	90.33 ± 4.16^b^	41.40 ± 0.90^a^	323.33 ± 11.50^a^
DMSA	8.37 ± 0.83^b^	6.80 ± 0.30^b^	0.33 ± 0.06^b^	1.07 ± 0.15^b^	7.22 ± 0.24^b^	124.67 ± 3.21^a^	43.40 ± 3.00^a^	370.33 ± 7.23^a^
ATX‐L	9.60 ± 0.82^b^	6.87 ± 1.86^b^	0.37 ± 0.06^b^	1.10 ± 0.10^b^	7.40 ± 0.24^b^	118.33 ± 5.51^a^	43.33 ± 3.88^a^	354.33 ± 15.57^a^
ATX‐H	7.97 ± 0.40^b^	6.13 ± 0.38^b^	0.40 ± 0.10^b^	1.23 ± 0.06^b^	7.98 ± 0.16^a^	112.33 ± 4.04^a^	42.43 ± 3.88^a^	346.67 ± 8.33^a^

*Note:* The different letters (a–c) indicate significant differences (*p* < 0.05).

^a^ All values are expressed as the means ± SDs (*n* = 6). Mean separation was performed using Duncan's multiple range test.

### 3.3 Assessment of Kidney Function Indicators and Pathologic Changes in Pb‐Exposed Mice With or Without ATX Supplementation

**FIGURE 1:**
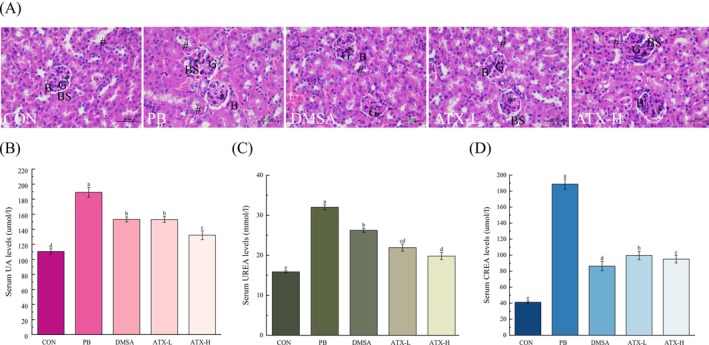
Effects of lead and ATX on kidney function and pathological changes. (A) Histological sections of mouse kidneys stained with H&E, showing sequentially from left to right the CON, Pb, DMSA, ATX‐L, and ATX‐H groups. H&E, Hematoxylin and eosin staining; scale bar = 50 μm (×400 magnification); B, Bowman's capsule; BS, Bowman's space; G, Glomerulus; *: Mesangial cells; #: Distal convoluted tubule. (B) UA levels, (C) Urea levels, (D) CREA levels. Values are expressed as means ± standard deviations (SD) (*n* = 8). Different letters (a–e) indicate significant differences (*p* < 0.05) according to Duncan's multiple range test in ANOVA for group comparisons.

Control group kidneys showed intact structures (Figure 1A), including the proximal convoluted tubule, distal convoluted tubule (#), Bowman's capsule (B), Bowman's space (BS), and glomeruli (G). In contrast, the Pb‐treated group showed significant kidney structural abnormalities, including congestion, inflammatory infiltration, necrosis, and hemorrhage, a degenerated glomerulus with wide Bowman's space (G), and a detached basement membrane. There was also severe degeneration and necrosis in renal parenchymal cells, along with chronic inflammatory cell infiltration, high interstitial congestion around the glomerulus, dilation in interstitial blood vessels, wide lumens in distal tubules (#), and deformed proximal tubules with detached brush borders in the Pb‐treated group. The Pb‐treated groups without ATX had high damage scores for TD (3.65), TN (3.68), TIN (3.82), and THS (7.38), which were categorized as severe (Table 1). However, ATX‐supplemented Pb‐intoxicated mice exhibited a marked reduction in these pathological changes, with a restoration of renal architecture and low damage scores of TD (1.72, 1.5), TN (1.72, 1.57), TIN (1.54, 1.33), and THS (3.36, 2.92), which were graded as mild (Table 1).

The present results indicate that ATX treatment provides significant protective effects against renal impairment in lead‐intoxicated mice. Specifically, serum levels of uric acid (UA) (Figure 1B), urea (UREA) (Figure 1C), and creatinine (CREA) (Figure 1D) were substantially elevated in the lead exposure group (Pb), showing increases of 71.19%, 101.91%, and 358.67%, respectively, compared to the control group (CON), which suggests pronounced renal dysfunction due to lead exposure. In contrast, the positive control group (DMSA) and both ATX treatment groups (ATX‐L and ATX‐H) demonstrated significant reductions in these markers (*p* < 0.05). Notably, ATX‐H showed stronger reductions in UA and CREA levels compared to ATX‐L, while no statistically significant difference was observed between ATX‐L and ATX‐H in UREA levels. These findings suggest that ATX exerts substantial renoprotective effects, particularly in lowering UA and CREA, though the dose‐dependent trend for UREA was not significant.

### 3.4 Assessment of Liver Function Indicators and Pathologic Changes in Pb‐Exposed Mice With or Without ATX Supplementation

**FIGURE 2:**
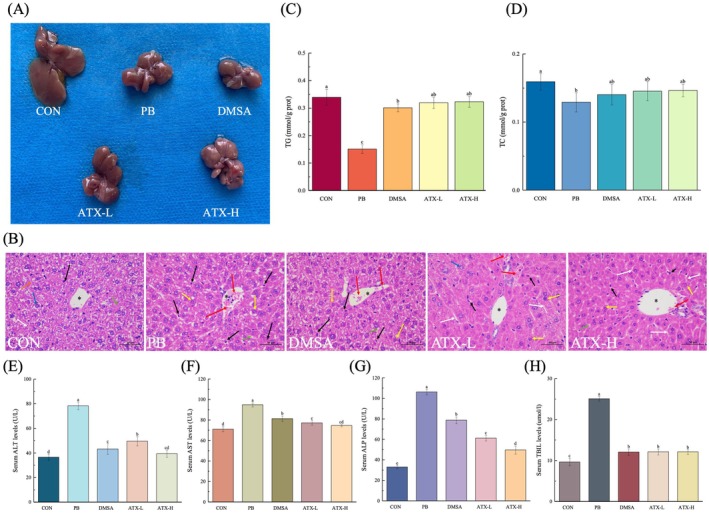
Effects of astaxanthin supplementation on liver function. (A) Liver samples. (B) Histological sections of mouse liver stained with H&E, shown sequentially from left to right as CON, Pb, DMSA, ATX‐L, and ATX‐H groups. H&E: Hematoxylin and eosin staining, scale bar = 50 μm (×400 magnification). Blue represents liver cells. Red represents dilation and congestion (blockage). White represents hepatic sinus blood. Orange represents liver plates. Yellow represents chromatin condensation, and black represents necrotic cells. (C) TG. (D) TC. (E) ALT. (F) AST. (G) ALP. (H) TBIL. Values are expressed as means ± standard deviation (SD) (*n* = 8). Different letters (a–e) indicate significant differences (*p* < 0.05) according to Duncan's multiple range test in ANOVA for group comparisons.

Histological analysis of liver tissue from the control group (Figure 2B) revealed intact hepatic architecture, normal central veins, and hepatocytes without any evident morphological abnormalities. In contrast, the lead‐exposed group showed disrupted hepatic parenchyma, damaged central veins, cytoplasmic vacuolation, portal hypertension, edema‐induced sinusoidal obstruction, and peripheral hepatocyte degeneration with vacuolation. Additionally, neutrophil and lymphocyte infiltration around the portal veins was observed in the Pb‐treated group, with a severe injury score of 3.84. ATX treatment significantly alleviated these pathological changes, providing notable hepatoprotection by preserving liver tissue integrity, reducing the tissue injury score (1.55 and 1.46), and maintaining a normal structural grade (Table 1).

ATX significantly improved liver function indicators in lead‐intoxicated mice. In the lead‐exposed group (Pb), triglyceride (TG) and total cholesterol (TC) levels were reduced by 37% and 39.07%, respectively (*p* < 0.05), indicating lipid metabolism disruption (Figure 2C,D). ATX‐H notably increased TG and TC by 51% and 53.87%, respectively. Additionally, ALP, ALT, and AST were markedly elevated in the Pb group by 221.91%, 114.03%, and 33.38%, respectively, compared to the control, indicating hepatocyte damage (Figure 2E–H). ATX‐H treatment reduced ALP, ALT, and AST levels by 53.29%, 49.66%, and 21.34%, respectively. Furthermore, compared to the Pb‐free group, TBIL levels rose by 159.55% in the Pb group and decreased by 51.75% in ATX‐H treatment, with TBIL levels close to the normal levels. However, for AST and TBIL, although numerical decreases were observed in ATX‐H treatment, the differences between ATX‐L and ATX‐H were not statistically significant (*p* > 0.05). In summary, ATX significantly mitigated lead‐induced hepatotoxicity through multi‐pathway regulation of lipid metabolism, liver enzyme activity, and bilirubin metabolism, with the high dose of ATX showing the most pronounced effects.

### 3.5 Effects of ATX Treatment on Oxidative Stress Markers in the Liver of Pb‐Exposed Mice

**FIGURE 3:**
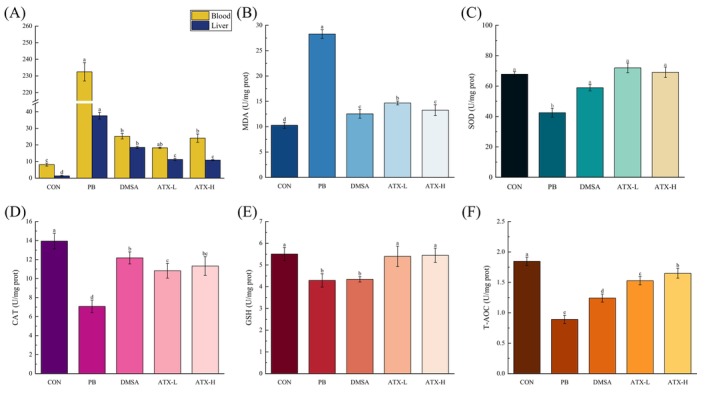
Effects of astaxanthin supplementation on oxidative stress indexes in liver of lead‐exposed mice. (A) After 8 weeks of lead exposure (end of all experiments), lead levels in the blood and liver of each group of mice, *n* = 8. (B) MDA levels in liver. (C) SOD levels in liver. (D) CAT levels in liver. (E) GSH levels in liver. (F) T‐AOC levels in liver. Values are expressed as means ± SD of triplicate (*n* = 8). The different letters (a–e) indicate significant differences (*p* < 0.05) according to Duncan's multiple range test in ANOVA, which are used for comparison among groups.

This study demonstrates that ATX significantly mitigates oxidative stress‐induced liver damage in lead‐intoxicated mice, exerting multi‐faceted hepatoprotective effects. At the end of the experiment, blood lead levels in the lead‐exposed mice were significantly elevated, with 28.40 times higher than those in the control group. Compared to the control group, Pb exposure increased hepatic Pb deposition by 27.54 times. Astaxanthin supplementation significantly reduced Pb accumulation in both liver and blood in the lead‐exposed mice (Figure 3A). Additionally, these markers reflect internal Pb burden after lead exposure. Results from Figure S2 indicate that Pb exposure increases lead levels in whole blood and liver tissue, while ATX supplementation alleviates blood Pb levels and weight loss. Notably, compared to the DMSA group, both ATX‐L and ATX‐H groups showed better efficacy in reducing blood Pb levels and mitigating weight loss in the lead‐exposed mice.

Tunel staining analysis (Figure S2F) showed a marked increase in apoptotic cells in the liver tissue of the Pb‐exposed group (Pb), whereas ATX, particularly in the high‐dose group (ATX‐H), significantly suppressed apoptosis, indicating that ATX has a pronounced inhibitory effect on lead‐induced apoptosis. Regarding oxidative stress markers, Pb exposure resulted in a significant increase in malondialdehyde (MDA) levels in liver tissue (Figure 3B), rising by 175.37% compared to the CON group. Concurrently, the activities of antioxidant enzymes superoxide dismutase (SOD) and catalase (CAT) were significantly reduced in the Pb group (Figure 3C,D), by 37.35% and 49.28%, respectively. Under ATX intervention, especially in the high‐dose group, MDA levels significantly decreased, restoring 53.11%, while SOD and CAT activities rebounded by 62.61% and 37.53%, respectively, indicating that ATX enhanced the endogenous antioxidant system. Furthermore, Pb exposure significantly reduced glutathione (GSH) content and total antioxidant capacity (T‐AOC) (Figure 3E,F), with decreases of 22.04% and 51.74%, respectively, compared to the CON group. However, in the ATX‐H intervention group, GSH and T‐AOC levels were significantly restored nearly to the level in the control group, reaching 26.96% and 85.13%, respectively. In summary, ATX effectively alleviates lead‐induced hepatic oxidative stress, likely by inhibiting lipid peroxidation, restoring antioxidant enzyme activity, and enhancing endogenous antioxidant reserves, thus providing substantial protective effects on hepatocyte apoptosis and overall antioxidant capacity recovery. It should be noted that although SOD and CAT activities were markedly restored in both ATX‐treated groups, no statistically significant difference was found between ATX‐L and ATX‐H (*p* > 0.05). A similar trend was observed for GSH levels, suggesting that although ATX enhanced antioxidant capacity, its dose‐dependent effects on enzymatic antioxidants were limited.

### 3.6 Transcriptomic Analysis of Liver Tissue in the Treated Mice

RNA‐seq of 15 liver samples yielded 115.65 Gb of clean data, with > 6 Gb of clean data per sample. Q30 values exceeded 92%, and the GC content ranged from 39.24% to 44.08%, with an overall sequencing error rate as low as 0.03%. Thus, sequencing quality was deemed high (Table S3), making the data suitable for subsequent analyses.

**FIGURE 4:**
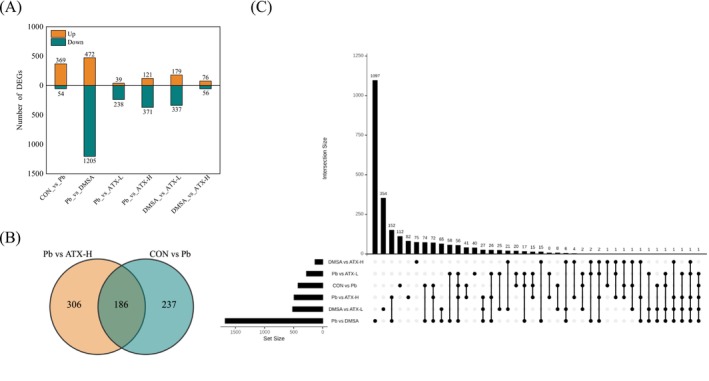
DEGs of liver in each treatment group of mice exposed to lead. (A) Number of up‐regulated and down‐regulated DEGs. The yellow symbolizes up‐regulation, and the green symbolizes down‐regulation. (B) Venn analysis of DEGs in CON vs. Pb and Pb vs. ATX‐H. (C) An upset diagram. The column on the left indicates the number of DEGs in different pairwise comparisons. The column on the right indicates the overlapping and accession‐specific DEGs among the different comparisons.

A comparative cross‐analysis of differential expressed genes (DEGs) across all liver samples under different treatments identified 3009 DEGs. In the comparison between CON and Pb, the number of upregulated DEGs exceeded downregulated genes. Conversely, in the comparisons of Pb vs. DMSA, Pb vs. ATX‐L, and Pb vs. ATX‐H, upregulated genes were lower than downregulated (Figure 4A). This analysis suggests that astaxanthin supplementation results in more stable gene expression compared to DMSA under lead exposure. A Venn diagram revealed 186 shared DEGs between CON vs. Pb and Pb vs. ATX‐H, with 237 DEGs uniquely expressed in CON vs. Pb and 306 DEGs uniquely expressed in Pb vs. ATX‐H (Figure 4B). Additionally, 57 DEGs were observed across all four comparison groups: CON vs. Pb, Pb vs. ATX‐H, Pb vs. ATX‐L, and Pb vs. DMSA (Figure 4C).

#### 3.6.1 KEGG Pathway and GO Enrichment Analysis

**FIGURE 5:**
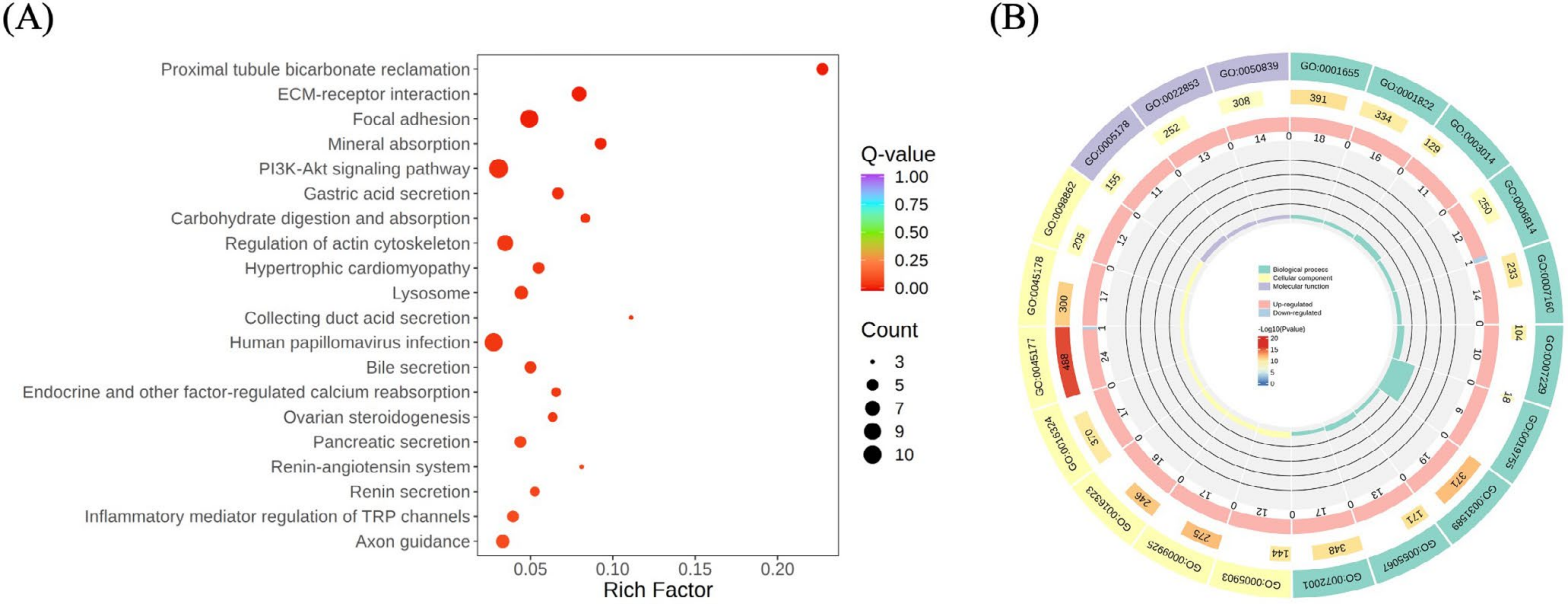
KEGG and GO analysis of DEGs. (A) KEGG analysis of 186 shared DEGs in Figure 5B. (B) GO analysis of 186 shared DEGs in Figure 5B. Green represents biological processes (BP), yellow represents cellular components (CC), and purple represents molecular function (MF). The outer circle represents the top 20 GO terms. The middle circle represents the gene count in the genomic background and the *p*‐value of gene enrichment for the specified GO term. The inner circle represents the number of DEGs. Pink represents an upward adjustment, and blue represents a downward adjustment.

KEGG and GO enrichment analyses of 186 shared DEGs between CON vs. Pb and Pb vs. ATX‐H revealed significant pathways and functional categories. Among the top 20 KEGG pathways, four had *p*‐values < 0.0001, including proximal tubule bicarbonate reclamation, ECM‐receptor interaction, focal adhesion, and mineral absorption, suggesting mechanisms like acid–base balance regulation, ECM–cell interaction, cytoskeletal connections, signal transduction, and mineral uptake under lead exposure (Figure 5A). GO enrichment analysis highlighted key subcategories in biological processes (BP) (e.g., cell–substrate adhesion, urogenital system development, monovalent inorganic cation homeostasis), molecular function (MF) (e.g., integrin binding, transmembrane transporter activity), and cellular components (CC) (e.g., apical plasma membrane, brush border, basal plasma membrane) (Figure 5B).

Genes uniquely upregulated in CON vs. Pb were enriched in aldosterone‐regulated sodium reabsorption, while genes altered in Pb vs. ATX‐H were enriched in pathways like biosynthesis of amino acids, carbon metabolism, PPAR signaling, and fatty acid metabolism (Figure S3). GO analysis showed that upregulated DEGs in CON vs. Pb were linked to ECM organization, metal ion homeostasis, and cell adhesion, while those in Pb vs. ATX‐H were related to kidney development, metabolic processes, and temperature regulation.

#### 3.6.2 qRT–PCR Verification of RNA–Seq Data

**FIGURE 6:**
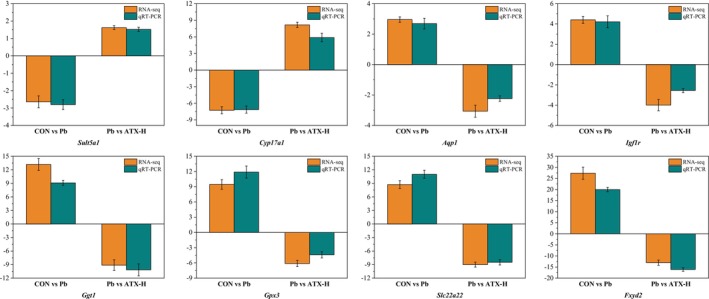
Comparison between the results of RNA‐seq and qRT–PCR of eight selected DEGs. The expression levels determined by the two methods were log_2_ transformed. Values represent mean ± standard deviation (SD) (*n* = 3).

To validate RNA‐seq findings, eight representative DEGs involved in steroid metabolism, antioxidant defense, cell repair and transport functions, and gut–liver axis regulation were selected for qRT‐PCR analysis. The results showed that the expression trends of these genes were consistent with those of RNA‐seq, confirming the reliability of the RNA‐seq results (Figure 6).

### 3.7 Metabolomic Analysis of Liver Tissue in the Treated Mice

**FIGURE 7:**
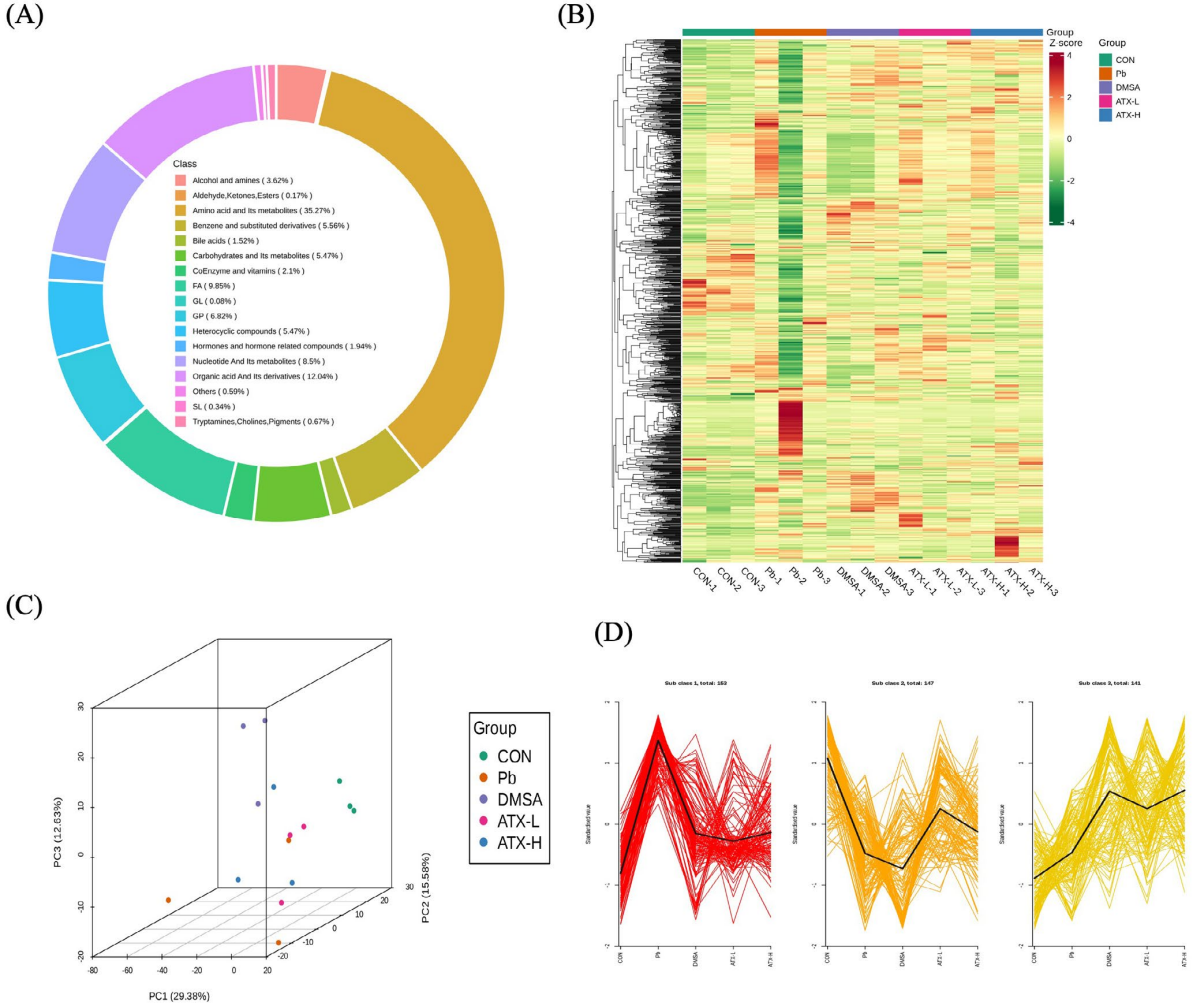
Identification of mouse liver metabolites. (A) Metabolite classes. (B) Stratified clustering heat maps of metabolites in all samples. (C) PCA score maps. (D) *K*‐means analysis.

Metabolomics analysis revealed the multi‐layered regulatory role of ATX in mitigating Pb‐induced hepatic metabolic imbalance in mice (Figure 7). A total of 441 metabolites were identified across 17 metabolic categories among the treatment groups (CON, Pb, DMSA, ATX‐L, ATX‐H) (Figure 7A). Hierarchical clustering heatmap analysis indicated distinct metabolic expression patterns between the lead exposure group and the control, with increased levels of lipid metabolism and oxidative stress‐related metabolites in the Pb group, which were restored toward normal levels by ATX‐H. This suggests that ATX may mitigate lead‐induced metabolic imbalance by modulating key metabolites (Figure 7B). Principal component analysis (PCA) further supported this observation, showing a marked metabolic profile shift in lead‐exposed mice away from the control group, with ATX, particularly at high doses, realigning the profile closer to the control, indicating ATX's role in restoring liver metabolic balance (Figure 7C). K‐means clustering analysis (Figure 7D) confirmed the abnormal enrichment of specific metabolite clusters, notably lipid metabolism and oxidative stress‐related clusters in the Pb group, with ATX‐H significantly suppressing this aberrant expression, underscoring ATX's ability to modulate hepatic metabolic pathways.

**FIGURE 8:**
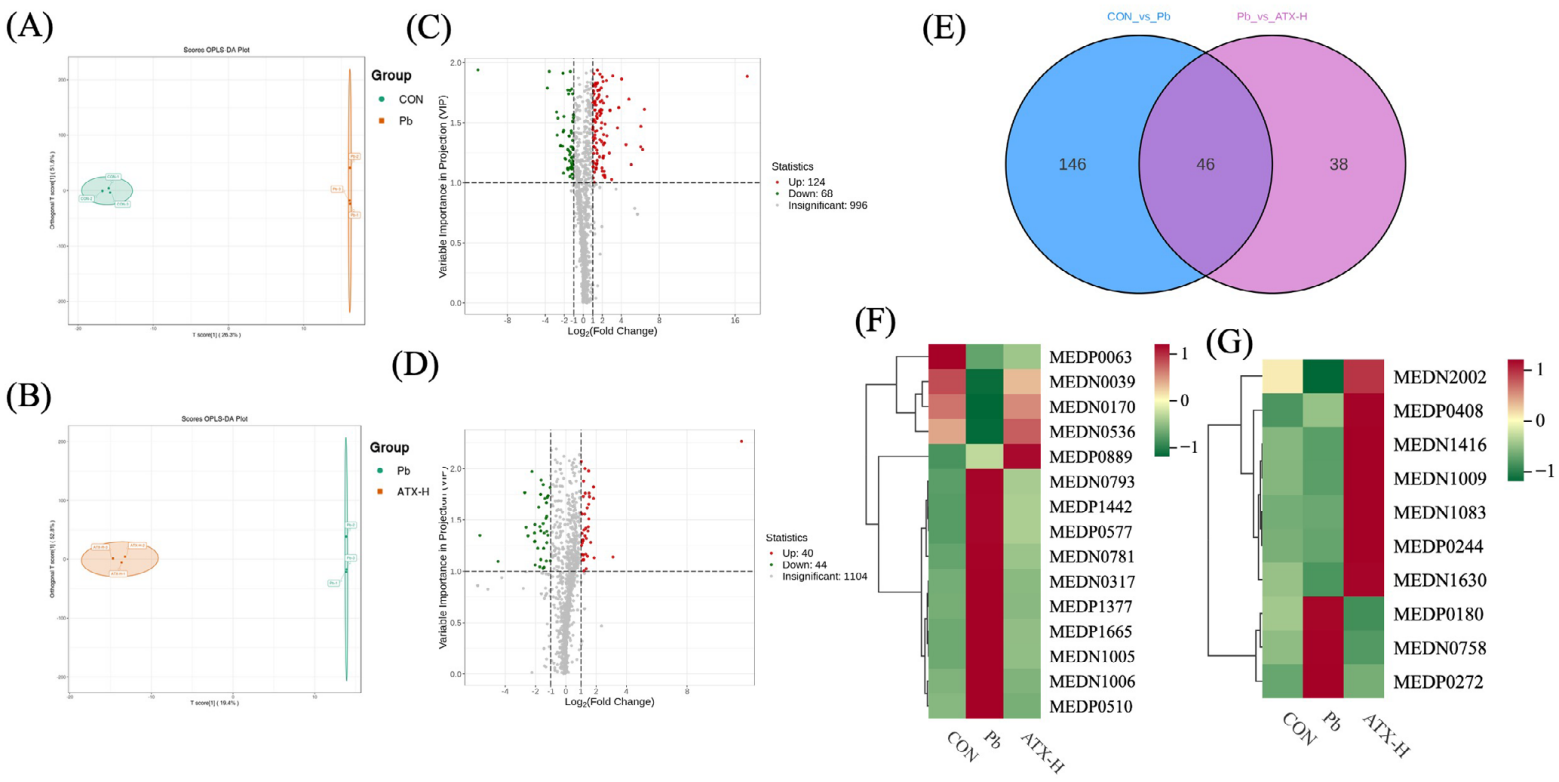
Differential metabolites species analyzed in CON vs. Pb group and Pb vs ATX‐H group. OPLS‐DA scores plots (A, B) and volcano plot (C, D) analysis of CON vs. Pb group and Pb vs. ATX‐H group, respectively. (E) Venn analysis of DEMs in CON vs. Pb and Pb vs. ATX‐H. (F) Typical metabolites in the DEMs of CON vs. Pb and Pb vs. ATX‐H groups. (G) Typical metabolites only in the DEMs of Pb vs. ATX‐H groups.

Further metabolomic analysis (Figure 8) elucidated ATX's significant role in ameliorating lead‐induced hepatic metabolic disorders. The OPLS‐DA score plots (Figure 8A,B) showed a clear separation between the Pb and control (CON) groups in metabolic profiles, reflecting substantial metabolic alterations caused by lead exposure (Figure 8A), while ATX‐H partially reversed these changes, with the metabolic profile moving closer to that of the control, further supporting ATX's effectiveness in restoring metabolic balance (Figure 8C). Volcano plots (Figure 8C,D) illustrated the significant metabolite changes between CON vs. Pb and Pb vs. ATX‐H, highlighting the regulation trends of key metabolites post‐lead exposure. Differential metabolite analysis (Figure 8E) revealed that lead exposure significantly impacted multiple metabolic pathways, with ATX‐H prominently regulating pathways associated with lipid metabolism, amino acid turnover, and antioxidant defense. In summary, ATX demonstrated substantial multi‐level liver protective potential by modulating metabolite distribution, restoring core metabolic patterns, and reshaping metabolic clusters.

In this study, significantly altered differential metabolites were screened based on Fold Change (FC) values and VIP values (data shown in Tables S4–, S6), and metabolites closely related to the hepatic metabolic response to lead exposure in mice were selected according to their functional roles and pathway associations. Figure 8F illustrates that lead exposure induced multiple metabolic pressures in the liver, which responded by enhancing fatty acid oxidation (Carnitine C20:1‐OH, Carnitine C2:0, Carnitine C3:0, Carnitine C4:0, Carnitine isoC4:0), glycolysis (Phosphoenolpyruvate), and detoxification metabolism (Hippuric Acid, Uric acid), accompanied by elevated levels of inflammation‐associated metabolites such as 6‐keto‐PGF1α and Prostaglandin E2. These changes suggest an adaptive response by the liver to increase energy supply and detoxification under lead exposure, although the resulting oxidative stress and inflammatory responses further exacerbated the metabolic burden. Meanwhile, the significant decrease in Uridine 5′‐Diphosphate, estrone 3‐sulfate, Cortisol, Glutathione Reduced form, and N‐Acetylaspartate indicates that Pb exposure impaired hepatic antioxidant defense, neuroprotection, and endocrine regulation. These metabolite changes reveal a disruption in multi‐layered hepatic metabolic balance due to lead exposure, increasing metabolic pressure and compromising multi‐system defenses.

The trends of differential metabolites shown in Figure 8G demonstrated the multifaceted protective effects of ATX in mitigating lead exposure toxicity. Importantly, the notable increase in antioxidant metabolites such as 11‐Cis‐Retinol, All‐Trans‐13,14‐Dihydroretinol, Ferulic Acid, and 9 (S)‐HOTrE indicates that ATX enhances hepatic antioxidant capacity via activation of the Nrf2/ARE (Nuclear factor erythroid 2–related factor 2/Antioxidant Response Element) signaling pathway, playing a critical role in membrane protection. Additionally, the decrease in (±)4‐HDHA reflects reduced membrane oxidative damage. Moreover, the modulation of Carnitine series metabolites may be related to the activation of the PPAR (peroxisome proliferator‐activated receptor) pathway, which is crucial in lipid metabolism and fatty acid oxidation, suggesting ATX's regulatory ability on hepatic lipid metabolism. Furthermore, the decrease in 5‐Hydroxyindole‐3‐Acetic Acid implies reduced neurotransmitter metabolism demand, suggesting ATX's efficacy in alleviating neurotoxicity stress due to lead exposure, thereby enhancing neuroprotection. Concurrently, the decrease in β‐Nicotinamide Mononucleotide (NMN) suggests reduced liver energy demand, as ATX increases metabolic efficiency and decreases NAD+ consumption. Finally, the increase in N‐Acetyl‐L‐Glutamic Acid and Sphingosine 1‐phosphate (S1P) indicates the activation of the S1P/S1PR pathway, facilitating detoxification and anti‐inflammatory signaling in the liver and further promoting adaptive responses in a toxic environment. These multi‐layered metabolic regulations underscore ATX's significant protective effects in mitigating lead exposure toxicity.

### 3.8 A Comprehensive Transcriptomic and Metabolomic Analysis to Examine the Effects of ATX Supplementation on the Liver of Pb‐Exposed Mice

The KEGG enrichment plots comparing CON vs. Pb and Pb vs. ATX‐H illustrate the connection between the metabolic disturbances induced by lead exposure and the restorative mechanisms of ATX intervention. Enrichment analysis of the control vs. lead‐exposed group (CON vs. Pb) revealed that lead significantly activated the “glutathione metabolism” pathway, along with notable disturbances in lipid metabolism pathways like “fatty acid degradation” and “glycerophospholipid metabolism”. These alterations reflect oxidative stress and lipid peroxidation burden caused by lead toxicity. Dysregulated interactions among these pathways further impaired amino acid metabolism and energy supply, particularly in key metabolic pathways such as alanine, aspartate, and glutamate metabolism, increasing cellular vulnerability to oxidative stress.

In contrast, enrichment analysis between the lead‐exposed and ATX groups (Pb vs. ATX‐H) highlighted ATX's multi‐faceted restorative effects on these metabolic imbalances. ATX not only enhanced “glutathione metabolism” to neutralize free radicals and reduce oxidative stress's impact on lipid metabolism but also significantly restored the “glycerophospholipid metabolism” pathway, promoting structural stability of the cell membrane. Notably, ATX's regulatory impact extended to amino acid pathways such as “alanine, aspartate, and glutamate metabolism,” providing additional reductive molecules and energy to support antioxidative defenses.

A comparison of the two enrichment plots demonstrates that ATX's effects are not limited to single‐pathway restoration but involve a synergistic effect among antioxidant, lipid, and amino acid metabolism pathways. This comprehensive regulatory mechanism helps the host to counteract the complex metabolic imbalances induced by lead exposure. Through its multi‐pathway regulatory effects, ATX not only alleviates oxidative stress but also stabilizes cell membrane structures and supports energy metabolism, facilitating the restoration of hepatic metabolic homeostasis. This cross‐pathway regulation underscores ATX's potential as a multi‐target protective agent against lead toxicity.

### 3.9 ATX Regulates Specific Gut Microbial Phyla in Pb‐Exposed Mice

As shown in Figure 10, ATX exhibited a multilayered modulatory effect on the gut microbiota of lead‐exposed mice, including restoration of community structure, increase in the proportion of beneficial bacteria, enhancement of microbial diversity, and mitigation of Pb‐induced dysbiosis. PCA and NMDS2 analyses (Figure 10A,B) showed that the microbial composition in the lead‐exposed group significantly deviated from the control, while the spatial distribution in the ATX‐treated groups gradually shifted closer to the control group, indicating partial restoration of gut structure by ATX. The Venn diagram (Figure 10C) reveals an increase in species unique to the ATX group, potentially enhancing community diversity via proliferation of beneficial bacteria or inhibition of harmful bacteria. Alpha diversity analysis (Figure 10D) demonstrated that lead exposure significantly reduced microbial diversity, while ATX treatment effectively restored microbial richness and balance as indicated by the Chao1 and Shannon indices.

At the phylum level (Figure 10E), lead exposure increased the proportions of *Bacteroidetes* and *Proteobacteria* while decreasing *Firmicutes* and *Actinobacteria*, a dysbiosis potentially linked to inflammation and impaired barrier function. ATX intervention balanced these phyla ratios, notably reducing *Proteobacteria* abundance. At the family level (Figure 10F), an increase in *Bacteroidaceae* and *Enterobacteriaceae* in the lead‐exposed group likely contributed to pro‐inflammatory metabolites, while reductions in *Lactobacillaceae* and *Ruminococcaceae* weakened anti‐inflammatory potential. Following ATX intervention, beneficial bacteria, including *Lactobacillaceae* and *Ruminococcaceae*, showed a marked increase, potentially supporting the production of short‐chain fatty acid (SCFA) and contributing to anti‐inflammatory effects, thus promoting gut health. Additionally, the increase in *Faecalibacteriaceae* further strengthened the anti‐inflammatory and antioxidative microenvironment. In summary, ATX exhibits potential to alleviate lead‐induced gut dysbiosis and support gut health by restoring microbial structure, increasing anti‐inflammatory beneficial bacteria (e.g., *Lactobacillaceae*, *Ruminococcaceae*, and *Faecalibacteriaceae*), and inhibiting pro‐inflammatory harmful bacteria.

Figure 11 shows ATX's notable multi‐level regulatory effects on the gut microbiota of lead‐exposed mice. The genus‐level heatmap (Figure 11A) demonstrates an increase in pro‐inflammatory genera (e.g., *Enterobacter* and *Bacteroides*) and a reduction in anti‐inflammatory beneficial genera (e.g., *Lactobacillus* and *Ruminococcus*) due to lead exposure, disrupting gut microbial balance. ATX‐H effectively restored these beneficial genera, indicating both suppression of harmful taxa and promotion of beneficial ones. LDA effect size analysis (Figure 11B) confirmed these key microbial shifts, revealing that ATX significantly promotes anti‐inflammatory bacterial proliferation while inhibiting lead‐induced harmful bacteria. In LEfSe analysis, comparing DMSA and ATX‐H groups (Figure 11C), the ATX‐H group exhibited significantly higher relative abundances of beneficial genera, particularly anti‐inflammatory *Lactobacillus* and *Ruminococcus*, suggesting that ATX might have a more favorable effect than DMSA in promoting specific beneficial bacteria, though further validation in larger cohorts is required. The comparison between the CON and Pb groups (Figure 11D) highlighted increased harmful bacteria such as *Enterobacter* and *Bacteroides* and decreased beneficial bacteria due to lead exposure. Whereas, the Pb vs. ATX‐H comparison (Figure 11E) demonstrated a significant increase in anti‐inflammatory genera (e.g., *Lactobacillus*, *Ruminococcus*, and *Faecalibacterium*) and a reduction in pro‐inflammatory genera. Overall, high‐dose ATX intervention effectively restored gut microbial balance by promoting anti‐inflammatory beneficial bacteria and inhibiting harmful bacteria proliferation, mitigating lead‐induced gut inflammation and dysbiosis. This comprehensive modulation of gut microbiota suggests ATX's potential as a gut‐protective agent and also as potentially superior to DMSA in restoring microbial balance. It should be noted that the gut microbiota data presented here are based on a small sample size (*n* = 3 per group), which limits the generalizability of the findings. Although the observed trends are biologically plausible and consistent with the expected effects of ATX, these results need further verification in studies with larger sample sizes.

### 3.10 Multi‐Omics Integrated Analysis Reveals the Multidimensional Regulatory Mechanisms of ATX in Hepatic Protection

An integrated analysis was conducted on key metabolites specific to astaxanthin (ATX) supplementation, differentially expressed genes, and significantly altered microbial taxa using Spearman correlation with (*r* > 0.8) and *p* < 0.05. The results (Figure 12) showed that key antioxidant metabolites, including ferulic acid and sphingosine 1‐phosphate, were upregulated and strongly correlated with lipid metabolism genes (e.g., *Scd1*, *Fasn*), thereby contributing to improved hepatic lipid metabolism and homeostasis. ATX also elevated N‐acetyl‐L‐glutamic acid levels, supporting glutathione metabolism and detoxification. Concurrently, ATX decreased levels of pro‐inflammatory metabolites (e.g., (±)4‐HDHA), thus alleviating oxidative stress. These metabolite‐level modulations contributed to a stabilization of hepatic metabolic homeostasis, mitigating the disturbances induced by lead exposure. Upregulation of lipid metabolism genes (e.g., *Scd1*, *Fasn*) facilitated fatty acid metabolism, contributing to membrane repair and antioxidant defense. Expression of the anti‐inflammatory marker *Angptl4* correlated strongly with antioxidant metabolites and beneficial bacteria (e.g., *Lactobacillus*), suggesting that ATX supports lipid metabolism and anti‐inflammatory pathways, thereby attenuating hepatic inflammation. Furthermore, ATX suppressed the expression of pro‐inflammatory genes (e.g., *Ehhadh*), reducing inflammatory markers and oxidative stress, which collectively contribute to multi‐level genetic protection of liver health. ATX also induced marked changes in the gut microbial community, promoting beneficial bacterial growth (e.g., *Lactobacillus*, *Ruminococcus*) while inhibiting pro‐inflammatory taxa (e.g., *Desulfovibrio*, *Shigella*), thereby restoring a balanced gut microbiota. The increased abundance of beneficial bacteria enhanced the production of SCFA, which exerted anti‐inflammatory and antioxidant effects via the gut–liver axis, thereby supporting hepatic metabolic homeostasis. Concurrently, the reduction in pathogenic bacteria limited the accumulation of toxins and inflammatory mediators, further alleviating hepatic stress.

These findings highlight the gut–liver axis as a central mechanism in ATX‐mediated hepatic protection. Through gut microbiota modulation, metabolite regulation, and gene expression reprogramming, ATX exerts a systemic protective effect. SCFAs and other gut‐derived metabolites reinforce barrier integrity but also modulate hepatic metabolic and immune responses via the gut–liver axis, effectively mitigating oxidative stress and inflammation in the liver induced by lead exposure. This multi‐faceted regulatory mechanism underscores ATX's potential in hepatic protection and providing insights for potential therapeutic applications of ATX.

## 4 Discussion

Heavy metal pollution, especially lead (Pb), poses a serious threat to biological health and environmental safety (Zhao et al. 2022). This study, using a lead‐exposed mouse model, investigates the mitigating effects of astaxanthin (ATX) on lead toxicity. Results indicate that ATX confers multi‐level protection against lead‐induced damage. Specifically, ATX alleviates adverse effects such as weight loss, dyslipidemia, liver and kidney dysfunction, increased oxidative stress, and gut microbiota imbalance caused by lead exposure. In terms of physiological and biochemical indicators, ATX intervention effectively mitigated organ damage induced by lead. Metabolomic and transcriptomic analyses revealed that ATX regulates multiple key metabolic pathways, restoring oxidative stress and lipid metabolism balance. In the gut microbiome, ATX promoted the growth of anti‐inflammatory beneficial bacteria while inhibiting the increase of pro‐inflammatory harmful bacteria. These findings highlight ATX's protective effects, particularly at higher doses, suggesting its significant potential in the therapy of multi‐system damage induced by lead exposure.

### 4.1 ATX Improves Overall Health in Pb‐Exposed Mice

This study demonstrates that ATX significantly alleviates multiple adverse effects caused by lead toxicity. First, ATX improved lead‐induced weight loss in mice, which may be related to its regulatory effects on metabolic processes and promotion of energy balance (Xue et al. 2022). Furthermore, ATX exerts protective effects on the liver and kidneys, notably restoring lead‐altered liver and kidney weights and reducing tissue damage (Erbaş et al. 2024). In terms of lipid metabolism, ATX corrected lead‐induced reductions in total cholesterol (TC) and triglycerides (TG), indicating its important role in regulating lipid metabolism (Wang et al. 2019). Improvement in hematological parameters further supports ATX's protective effects, as ATX reversed lead‐induced decreases in red blood cell counts and hemoglobin concentration, as well as increases in white blood cell and lymphocyte counts, potentially due to its antioxidative, anti‐inflammatory, and immune‐modulatory properties (Ma et al. 2022). Compared to the traditional chelating agent meso‐2,3‐dimercaptosuccinic acid (DMSA), ATX displayed better safety, as no significant side effects were observed while protecting multiple organ systems (Flora et al. 2022). These results align with the known reports, further confirming ATX's potential application in the prevention and treatment of lead poisoning. Overall, ATX, through its diverse biological effects, effectively mitigates multi‐system damage induced by lead exposure, providing new insights into lead toxicity management.

### 4.2 ATX Ameliorates Lead‐Induced Renal Dysfunction in Mice

This study explored the protective effects of ATX on renal dysfunction induced by lead exposure. Mice in the lead‐exposed group exhibited significant pathological changes in renal tissue, manifesting as structural abnormalities and cellular damage related to lead‐induced oxidative stress and inflammation. Lead exposure induces ROS overproduction and lipid peroxidation, causing damage to renal cell membranes and DNA (Dkhil et al. 2016). As a potent antioxidant, ATX can scavenge excess ROS, reducing oxidative stress damage to the kidneys. Additionally, its anti‐inflammatory properties inhibit the release of pro‐inflammatory cytokines, minimizing inflammatory damage to renal tissue (Akca et al. 2018). After ATX treatment, the renal tissue structure of mice showed marked improvement, with the integrity of glomeruli and renal tubules restored, possibly due to ATX's upregulation of endogenous antioxidant enzyme activity, enhancing antioxidant defenses (Chen et al. 2020). Moreover, ATX reduced serum levels of uric acid, urea, and creatinine, improving glomerular filtration and tubular reabsorption. This effect may result from ATX's reduction of oxidative damage in the kidneys, improved renal blood flow, and enhanced microcirculation, promoting normal waste excretion (Li et al. 2020). The protective effects of ATX were dose‐dependent, with higher doses showing more pronounced results, suggesting that a higher ATX dose may further enhance renal protection (Rao et al. 2006). In contrast, while DMSA effectively promotes lead excretion, it may exert nephrotoxicity, limiting its clinical use (Kojima et al. 1991). With its natural and safe characteristics, ATX demonstrates greater potential for application in the treatment of lead‐induced damages (Avila‐Carrasco et al. 2021).

### 4.3 ATX Mitigates Liver Dysfunction in Pb‐Exposed Mice Through Multiple Mechanisms

This study explored the protective effects of ATX on liver dysfunction in lead‐exposed mice, revealing that ATX exerts robust hepatoprotective effects through multiple mechanisms. The liver tissues of the lead‐exposed group displayed severe pathological changes, such as disrupted hepatocyte alignment and central vein damage, indicating substantial structural and functional liver impairment (Offor et al. 2017). ATX treatment markedly improved these pathological alterations, restoring normal liver architecture and reducing damage scores. This improvement may be attributed to ATX's antioxidative and anti‐inflammatory effects, which mitigate lead‐induced cellular injury and inflammation (Laderian et al. 2024). Lead exposure significantly reduced serum levels of TC and TG, while ATX partially restored these levels, potentially by regulating the PPAR signaling pathway and promoting the expression of lipid metabolism‐related genes (Jia et al. 2016).

In addition, ATX significantly lowered serum liver enzymes (ALP, ALT, and AST), thereby preserving hepatocyte membrane integrity, possibly through the inhibition of lipid peroxidation (Cai et al. 2022). ATX also reduced TBIL levels, enhancing liver detoxification function, which may be linked to its effect on increasing the liver's capacity to process bilirubin (Ma et al. 2020). Oxidative stress plays a critical role in lead‐induced liver damage, and as a potent antioxidant, ATX reduces MDA levels, restores the activities of SOD and CAT and replenishes GSH content, thereby strengthening antioxidative defenses (Cui et al. 2020). ATX also significantly suppressed apoptosis, possibly by modulating apoptosis‐related signaling pathways to protect hepatocytes (Kanwugu and Glukhareva 2023). Moreover, ATX reduced lead levels in blood and liver tissues, likely by promoting lead excretion or binding to lead ions, thus lowering the body's lead burden (Vaziri et al. 1999).

### 4.4 ATX Regulates Liver Transcriptome to Cope With Pb‐Induced Toxicity in Mice

Liver transcriptome sequencing revealed that lead exposure caused abnormal gene expression in numerous biological processes, including oxidative stress, inflammatory response, cell adhesion, and metabolic dysregulation (Gautam et al. 2015). In the lead‐exposed group, the number of upregulated DEGs outnumbered downregulated ones, potentially exacerbating cellular dysfunction (Zhang et al. 2022). KEGG pathway analysis showed that these DEGs were enriched in pathways such as proximal tubule bicarbonate reclamation and extracellular matrix‐receptor interaction, suggesting that lead may impair renal function, disrupt cell structure, and interfere with mineral metabolism (Heering et al. 1996).

In our study, after ATX intervention, gene expression tended to return to normal, with downregulated genes outnumbering upregulated ones, indicating that ATX may suppress lead‐induced aberrant gene expression and restore gene homeostasis. Specific enriched pathways, such as the PPAR signaling pathway, amino acid biosynthesis, and carbon metabolism, are highly relevant to energy metabolism and antioxidative defense mechanisms. ATX may activate these pathways to enhance cellular antioxidative capacity and mitigate lead toxicity, which is consistent with the previous reports (Jia et al. 2012; Lin et al. 2017; Chou et al. 2016). GO functional analysis further supported these findings, suggesting that ATX may play a protective role by maintaining organ function, regulating metabolism, and preserving cellular structure, which is also supported by the previous work (Wang, Liu, et al. 2022; Luo et al. 2022).

### 4.5 ATX Alleviates Metabolic Disorders in Pb‐Exposed Mice

Metabolomic analysis in the present study revealed that ATX effectively ameliorated lead‐induced disruptions in lipid, amino acid, and nucleotide metabolism, and compromised the hepatic antioxidant defense system as reported previously (Li, Jia, et al. 2024; Li, Ke, et al. 2024; Yang et al. 2018). Lead exposure caused a significant increase in lipid peroxidation and oxidative stress markers, reflecting severe hepatic damage (Figures 2, 3). ATX intervention reduced these metabolites, likely by inhibiting lipid peroxidation through the activation of the Nrf2/ARE pathway, which enhances antioxidant defenses and mitigates ROS accumulation (Islam et al. 2017; Ma 2013). Additionally, ATX regulated nucleotide metabolism by reducing elevated nucleotide metabolites, thereby safeguarding genomic integrity (Zou et al. 2024). Principal component and clustering analyses (Figure 7) confirmed that ATX normalized the metabolic profile disrupted by lead exposure, reinforcing its hepatoprotective potential.

Lead exposure induced compensatory upregulation of fatty acid oxidation, glycolysis, and detoxification pathways, accompanied by oxidative stress and inflammation (Lee et al. 2019). Increased carnitine metabolites (Figure 3) highlighted energy demands, but prolonged β‐oxidation exacerbated mitochondrial ROS production and lipid peroxidation (Brenner et al. 2013). Enhanced glycolysis provided short‐term energy but contributed to long‐term metabolic dysregulation (Tang 2020). Elevated hippuric acid (Figure 2) indicated active detoxification but may deplete antioxidant reserves (Xu et al. 2024). ATX restored metabolic balance by activating the PPAR pathway (Figure 8), which stabilized lipid metabolism through genes like Scd1 and Fasn (Ide et al. 2003). It also reduced 5‐hydroxyindole‐3‐acetic acid (Figure 8), alleviating neurotoxic stress, and activated the S1P pathway to mitigate inflammation and promote hepatic repair (Zeng et al. 2023). These findings underscore ATX's systemic regulatory effects in alleviating lead‐induced metabolic and inflammatory disturbances.

### 4.6 ATX Mitigates Pb‐Induced Metabolic Imbalance

**FIGURE 9:**
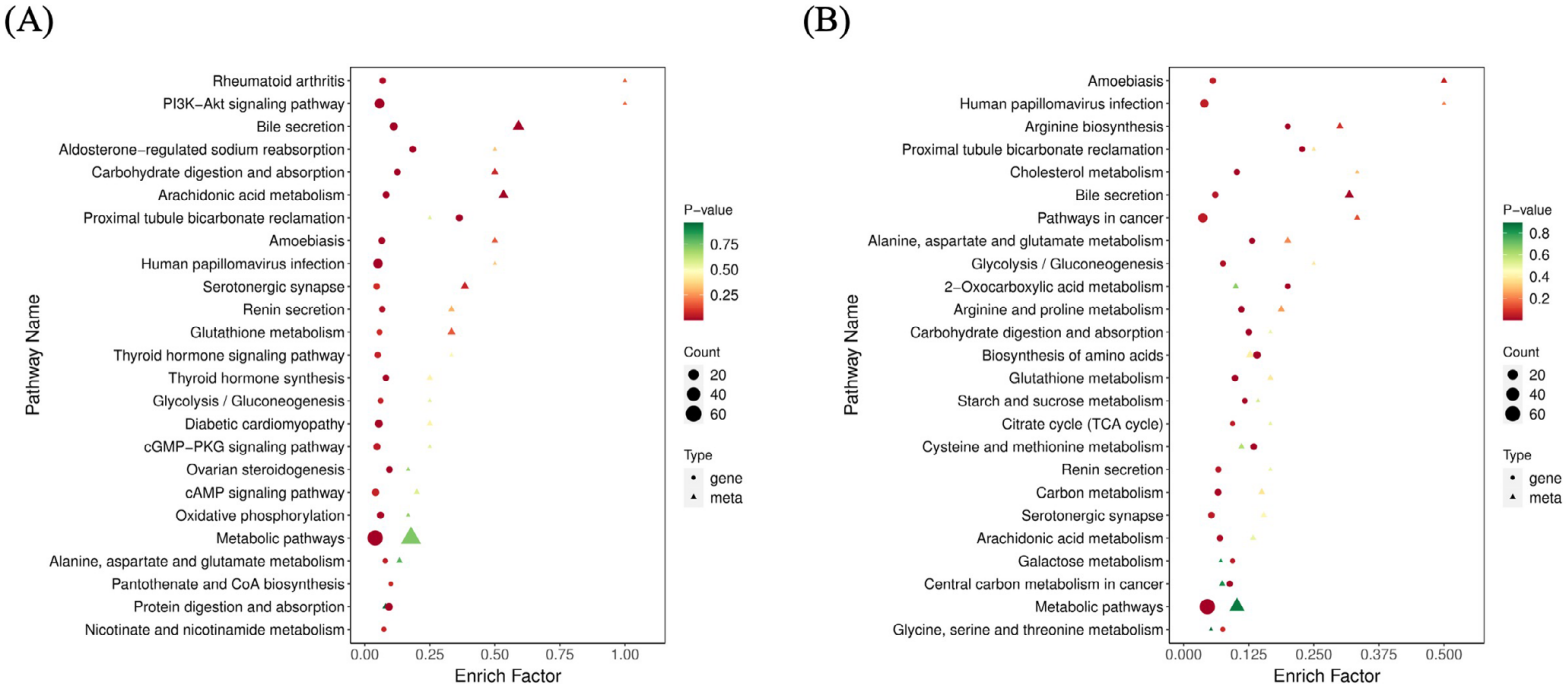
KEGG enrichment analysis of transcriptome and metabolome: (A) CON vs. Pb and (B) Pb vs. ATX‐H.

Integrated transcriptomic and metabolomic analyses (Figure 9) demonstrated that ATX significantly improved lead‐induced metabolic disturbances through synergistic, multi‐pathway regulation, highlighting its broad molecular regulatory potential. KEGG enrichment analysis (Figure 9) showed that lead exposure activated the “glutathione metabolism” pathway and disrupted key lipid metabolic pathways (e.g., fatty acid degradation, glycerophospholipid metabolism), thereby increasing susceptibility to oxidative stress. ATX intervention not only enhanced “glutathione metabolism,” neutralizing free radicals and reducing oxidative stress's impact on lipid metabolism but also restored the “glycerophospholipid metabolism” pathway, stabilizing cell membrane structures. ATX function in this regard was similar to that reported previously (Penugonda et al. 2006; Dkhil et al. 2016; Chen et al. 2024; Ledda et al. 2019).

Moreover, ATX regulated amino acid metabolism pathways by restoring key pathways such as alanine, aspartate, and glutamate metabolism (Figure 3), which provided cells with additional reducing agents and energy to support antioxidative defenses (Jiang et al. 2024). This multi‐layered regulatory mechanism spanning transcriptomic and metabolomic pathways highlights ATX's comprehensive protective potential against lead toxicity, enabling it to target complex metabolic disruptions caused by Pb toxicity. Compared to single‐pathway interventions, ATX's multi‐dimensional regulatory strategy is more effective in restoring metabolic homeostasis, underscoring its potential as a therapeutic agent for lead poisoning prevention and treatment.

### 4.7 EATX Ameliorates Gut Dysbiosis in Pb‐Exposed Mice by Modulating Gut Microbiota

**FIGURE 10:**
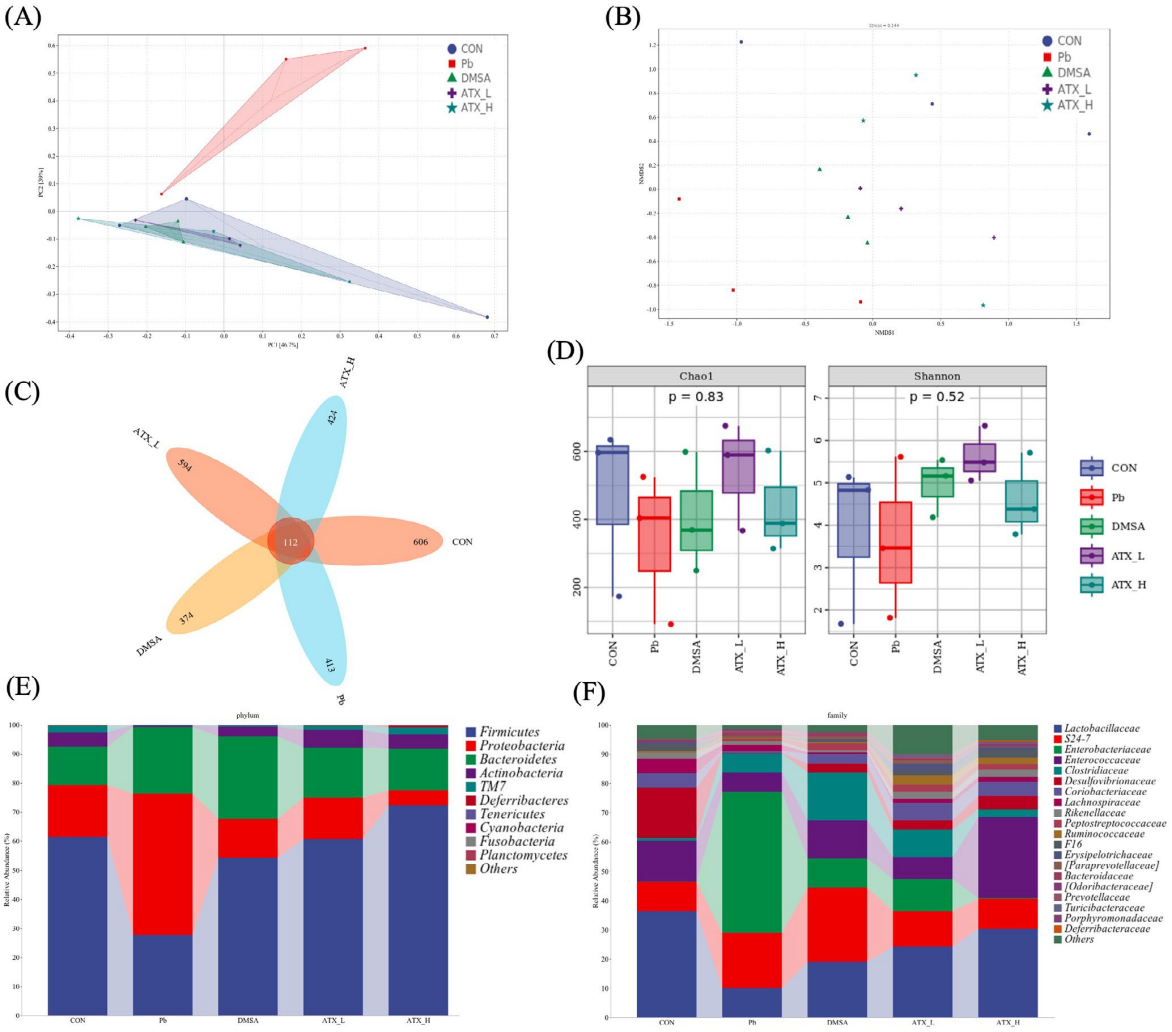
Changes of intestinal flora in lead‐exposed mice induced by astaxanthin supplementation. (A) PCA score maps. (B) NMDS2 analysis. (C) Venn diagram. (D) Alpha analysis of Chao1 and Shanon. (E) Composition of gut microbiota at the phylum level. (F) Composition of gut microbiota at the family level. Values are expressed as means ± SD of triplicate (*n* = 3).

**FIGURE 11:**
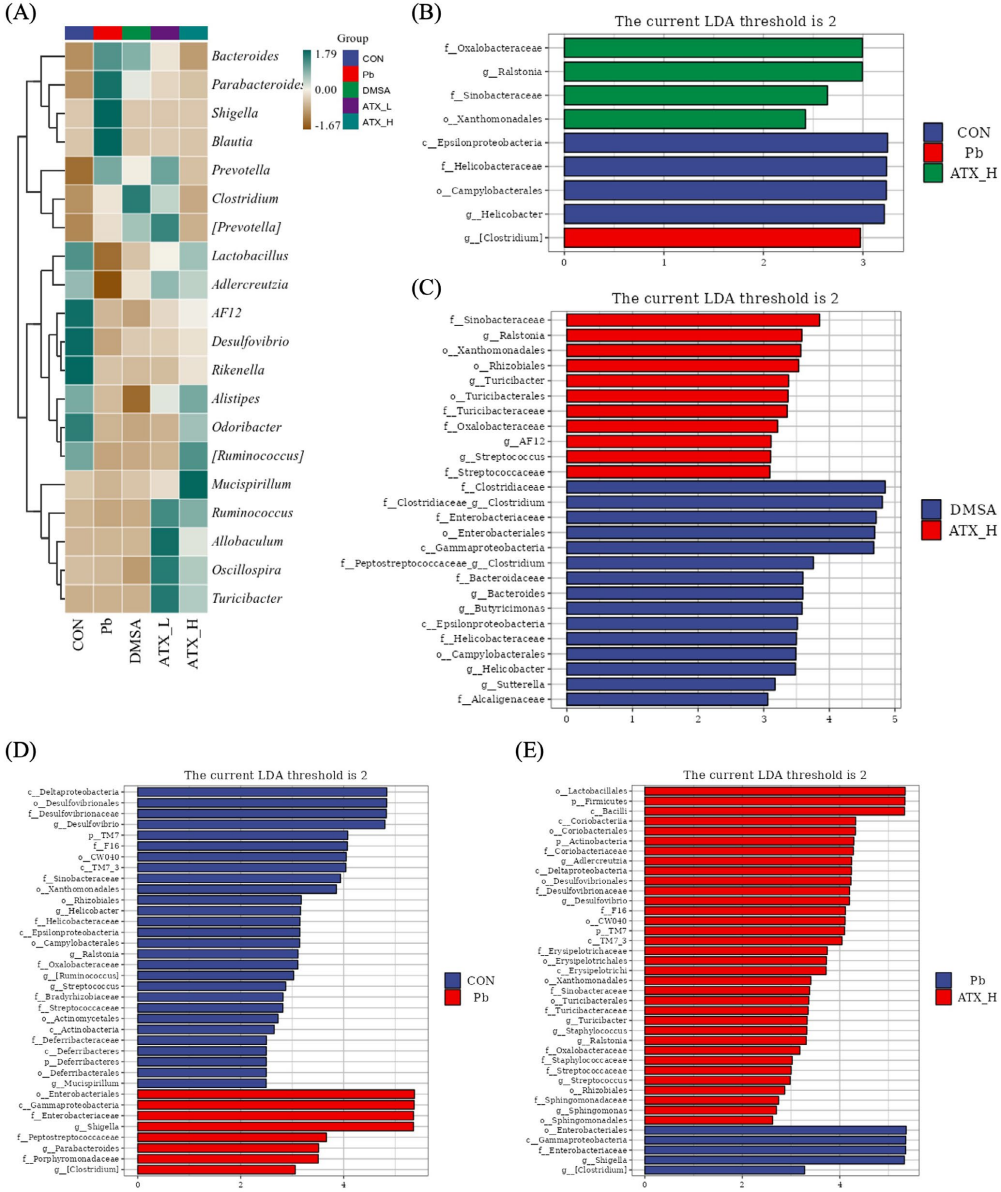
Analysis of microbial flora. (A) The species of composition heatmap based on genus‐level community and cluster analysis. (B) LDA effect size of overall classification of samples based on Kruskal–Wallis. LEfSe comparison of gut microbiota between DMSA and ATX‐H groups (C), CON and P*b* groups (D), P*b* and ATX‐H (E). Only taxa with a LDA score higher than 2 are listed.

This study reveals that ATX supplementation significantly restores gut microbiota dysbiosis caused by lead exposure in mice (Figure 10), highlighting the critical role of gut microbiota regulation in mitigating lead toxicity (Zeng et al. 2022). Lead exposure significantly reduced gut microbial diversity, decreasing the abundance of beneficial bacteria (such as *Lactobacillaceae* and *Ruminococcaceae*) while increasing harmful bacteria (such as *Enterobacteriaceae* and Bacteroidaceae) (Figure 10), disrupting gut microbial balance and potentially promoting inflammation and gut barrier dysfunction (Quaglio et al. 2022; Liu et al. 2020). ATX intervention, especially at high doses, effectively reversed these changes, bringing the gut microbiota structure closer to that of the control group. ATX increased the abundance of beneficial bacteria, promoting SCFA‐producing and anti‐inflammatory bacterial taxa while reducing the proportion of harmful pro‐inflammatory bacteria (Figure 11). These beneficial bacteria improve the gut environment, enhance barrier function, and reduce endotoxin absorption, potentially further alleviating lead‐induced systemic inflammation and oxidative stress (Li, Jia, et al. 2024; Li, Ke, et al. 2024; Wang, Li, et al. 2024; Silva Meneguelli et al. 2024; Song et al. 2022). Compared to the traditional chelating agent DMSA, ATX exhibited superior efficacy in modulating gut microbial homeostasis and promoting the growth of specific beneficial bacteria (Duan et al. 2020). This suggests that ATX offers dual protective effects by both modulating gut microbiota and reducing systemic Pb burden. In summary, ATX offers a novel approach to mitigate lead‐induced dysbiosis by multilayer regulation of the gut microbiota.

### 4.8 Integrative Mechanisms Linking Gut Microbiota, Metabolite Changes, and Hepatic Gene Expression

**FIGURE 12:**
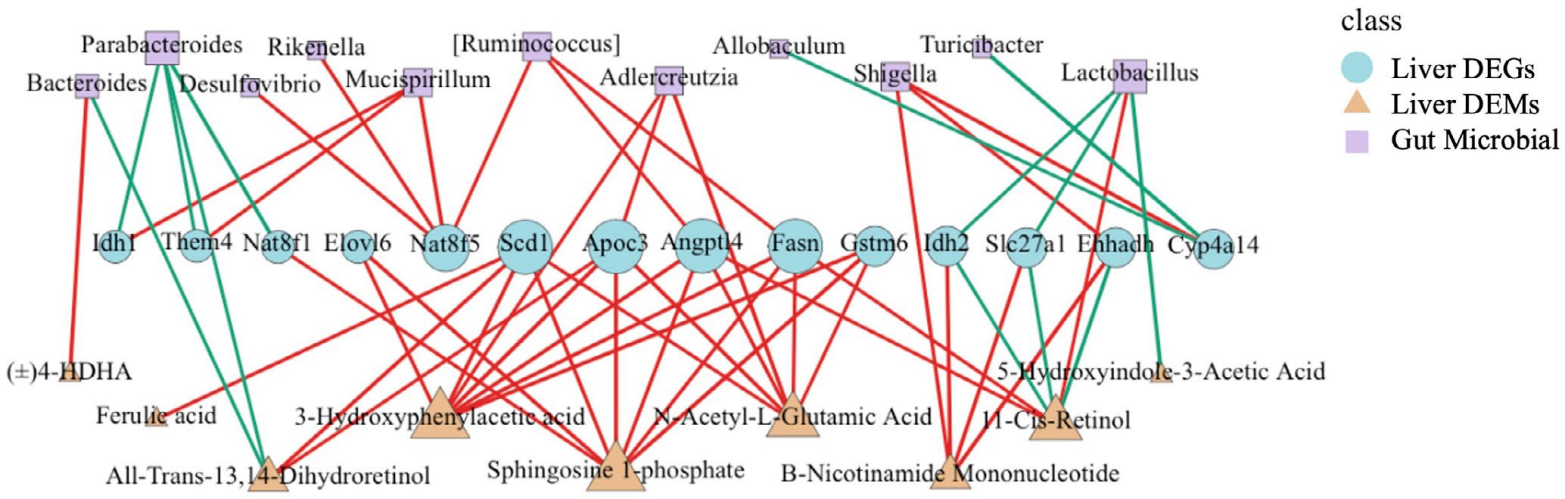
Multi‐omics correlation analysis of differential metabolites, genes, and gut microbiota in lead‐exposed mice following ATX intervention. Yellow triangles: Differential metabolites. Green circles: Differential genes. Purple squares: Gut microbiota. Positive correlations are represented by red lines, while negative correlations are depicted with green lines.

Our study reveals that the hepatoprotective effects of ATX against lead‐induced toxicity are mediated through a multi‐layered regulatory network involving gut microbiota modulation, metabolite remodeling, and hepatic gene expression regulation. ATX supplementation significantly increased the abundance of beneficial bacteria, such as *Lactobacillus* and *Ruminococcus*, while suppressing pathogenic genera, such as *Desulfovibrio* and *Shigella* (Figure 10), thereby restoring gut microbiota balance (Liu et al. 2018; Wu et al. 2025). The proliferation of SCFA‐producing bacteria likely contributed to the production of anti‐inflammatory and antioxidative metabolites. As reported in previous studies, SCFAs exert critical regulatory effects on hepatic immune and metabolic functions via the gut–liver axis (Yin et al. 2023). Our correlation analysis (Figure 12) revealed significant positive correlations between the abundance of beneficial bacteria and antioxidant metabolites, such as ferulic acid and sphingosine 1‐phosphate, as well as reduced pro‐inflammatory metabolites (e.g., (±)4‐HDHA). Concomitant with these metabolite changes, ATX also induced the upregulation of key lipid metabolism genes (e.g., *Scd1*, *Fasn*), which facilitated fatty acid synthesis and membrane repair, alleviating oxidative damage. Furthermore, ATX intervention enhanced “glutathione metabolism”, thereby neutralizing free radicals and reducing oxidative stress's impact on lipid metabolism. Additionally, ATX restored the “glycerophospholipid metabolism” pathway, contributing to the stabilization of cell membrane structures, thus closely linking lipid metabolism with oxidative stress regulation (Wang et al. 2022; Lin et al. 2025). Moreover, the expression of the anti‐inflammatory gene *Angptl4* strongly correlated with both antioxidant metabolites (e.g., ferulic acid) and beneficial bacteria (e.g., *Lactobacillus*) (*r* > 0.8, *p* < 0.05, as shown in Figure 12), suggesting its potential involvement in ATX‐mediated regulation of the gut–liver axis and its anti‐inflammatory effects (Albillos et al. 2020; Zhang et al. 2024). In summary, ATX exerts hepatoprotective effects through an integrated regulatory axis involving gut microbiota modulation, metabolite alteration, and hepatic gene expression, providing valuable mechanistic insights into its potential as a therapeutic agent for mitigating heavy metal toxicity.

## 5 Conclusion

This study demonstrates that ATX confers significant protective effects against lead‐induced toxicity through multi‐dimensional regulatory mechanisms. ATX effectively alleviated oxidative stress, inflammation, and metabolic disturbances by restoring redox homeostasis, regulating lipid, amino acid, and nucleotide metabolism, and activating key pathways such as Nrf2/ARE, PPAR, and S1P. These actions of ATX enhanced the antioxidant, anti‐inflammatory, and detoxification capacities of the host, preserving the structure and function of the liver and kidneys. Additionally, ATX restored gut–liver axis homeostasis by modulating the gut microbiota composition and promoting SCFA production, thereby reducing systemic inflammation and oxidative stress. These findings highlight ATX's potential as a safe and effective therapeutic agent for preventing and clinically treating heavy metal toxicity.

## Author Contributions

**Zhongyang Du:** data curation (equal), investigation (equal), software (equal), writing – original draft (equal). **Yan Sun:** data curation (equal), software (equal), writing – original draft (equal). **Xiaoli Zhu:** data curation (equal), software (equal), writing – original draft (equal). **Mengjing Liang:** investigation (equal), validation (equal). **Daming Shi:** investigation (equal), validation (equal). **Chunhui Zhang:** supervision (equal), writing – review and editing (equal). **Chunli Ji:** supervision (equal), writing – review and editing (equal). **Hongli Cui:** conceptualization (equal), funding acquisition (equal), resources (equal), supervision (equal). **Jinai Xue:** conceptualization (equal), funding acquisition (equal), resources (equal), supervision (equal). **Runzhi Li** **:** conceptualization (equal), funding acquisition (equal), resources (equal), supervision (equal), writing, review and editing (equal). **Xiaoyun Jia:** conceptualization (equal), funding acquisition (equal), resources (equal), supervision (equal), writing – review and editing (equal).

## Conflicts of Interest

The authors declare no conflicts of interest.

## Supporting information


**Figure S1:** Experimental protocol design.
**Figure S2:** Effects of ATX supplementation on body weight, food intake, and lipid levels, lead content in blood and urine in lead‐exposed mice. (A) Weight gain of mice recorded over the course of the experiment, *n* = 8; (B) Food intake, *n* = 8; (C) After 4 weeks (end of model building), lead levels in the blood and urine of each group of mice, *n* = 8; (D, E) Lipid profile in serum of mice, *n* = 8; TC, Total cholesterol; TG, Triglycerides. Values are expressed as means ± SD of triplicate (*n* = 8). The different letters (a–e) indicate significant differences (*p* < 0.05) according to Duncan's multiple range test in ANOVA, which are used for comparison among groups. (F) Tunel images of liver tissue.
**Figure S3:** KEGG and GO analysis of DEGs. (A) KEGG analysis of 237 shared DEGs in Figure 5B. (B) GO analysis of 237 shared DEGs in Figure 5B. (C) KEGG analysis of 306 shared DEGs in Figure 5B. (D) GO analysis of 306 shared DEGs in Figure 5B. In B and D, green represents biological processes (BP), yellow represents cellular components (CC), and purple represents molecular function (MF). The outer circle represents the top 20 GO terms. The middle circle represents the gene count in the genomic background and the *p*‐value of gene enrichment for the specified GO term. The inner circle represents the number of DEGs. Pink represents an upward adjustment, and blue represents a downward adjustment.

**Table S1:** Composition of experimental diet.

**Table S2:** Primers used for qRT–PCR.

**Table S3:** Quality evaluation of mouse liver transcriptome sequencing data.

**Table. S4** Identification of potential metabolite biomarkers both in CON vs. Pb and Pb vs. ATX‐H.

**Table. S5** Identification of potential exclusive metabolite biomarkers in CON vs. Pb.

**Table. S6** Identification of potential exclusive metabolite biomarkers in Pb vs. ATX‐H.

**Table S7:** Annotation of metabolites involved in the heat map in Figure 8.

## Data Availability

The data that support the findings of this study are available from the corresponding author upon reasonable request.
