# Supplementary material for: Astaxanthin Alleviates Lead‐Induced Toxicity by Restoring Hepatic and Gut–Liver Axis Homeostasis Through Multidimensional Metabolic and Antioxidative Pathways
Source: Food Sci Nutr. 2025 Sep 26;13(10):e70971. doi: 10.1002/fsn3.70971 (PMC12464569; doi:10.1002/fsn3.70971)
Supplement: Supplementary file 1 — Figure S1: Experimental protocol design. Figure S2: Effects of ATX supplementation on body weight, food intake, and lipid levels, lead content in blood and urine in lead‐exposed mice. (A) Weight gain of mice recorded over the course of the experiment, n = 8; (B) Food intake, n = 8; (C) After 4 weeks (end of model building), lead levels in the blood and urine of each group of mice, n = 8; (D, E) Lipid profile in serum of mice, n = 8; TC, Total cholesterol; TG, Triglycerides. Values are expressed as means ± SD of triplicate (n = 8). The different letters (a–e) indicate significant differences (p < 0.05) according to Duncan's multiple range test in ANOVA, which are used for comparison among groups. (F) Tunel images of liver tissue. Figure S3: KEGG and GO analysis of DEGs. (A) KEGG analysis of 237 shared DEGs in Figure 5B. (B) GO analysis of 237 shared DEGs in Figure 5B. (C) KEGG analysis of 306 shared DEGs in Figure 5B. (D) GO analysis of 306 shared DEGs in Figure 5B. In B and D, green represents biological processes (BP), yellow represents cellular components (CC), and purple represents molecular function (MF). The outer circle represents the top 20 GO terms. The middle circle represents the gene count in the genomic background and the p‐value of gene enrichment for the specified GO term. The inner circle represents the number of DEGs. Pink represents an upward adjustment, and blue represents a downward adjustment. [file FSN3-13-e70971-s006.pptx]

## Slide 1
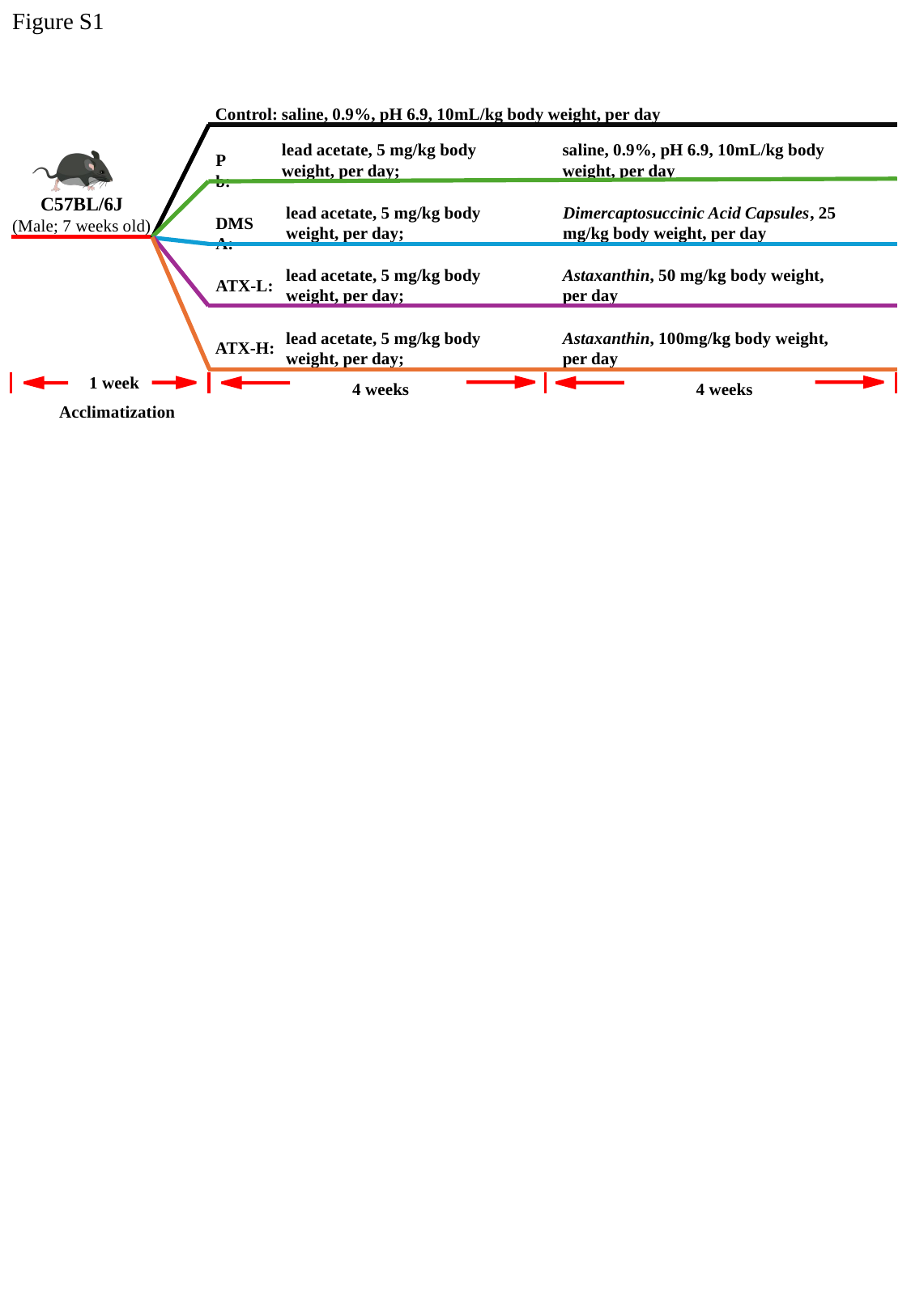

Figure S1
saline, 0.9%, pH 6.9, 10mL/kg body weight, per day
Control:
lead acetate, 5 mg/kg body weight, per day;
saline, 0.9%, pH 6.9, 10mL/kg body weight, per day
Pb:
C57BL/6J
(Male; 7 weeks old)
Dimercaptosuccinic Acid Capsules, 25 mg/kg body weight, per day
lead acetate, 5 mg/kg body weight, per day;
DMSA:
lead acetate, 5 mg/kg body weight, per day;
Astaxanthin, 50 mg/kg body weight, per day
ATX-L:
lead acetate, 5 mg/kg body weight, per day;
Astaxanthin, 100mg/kg body weight, per day
ATX-H:
1 week
4 weeks
4 weeks
Acclimatization

## Slide 2
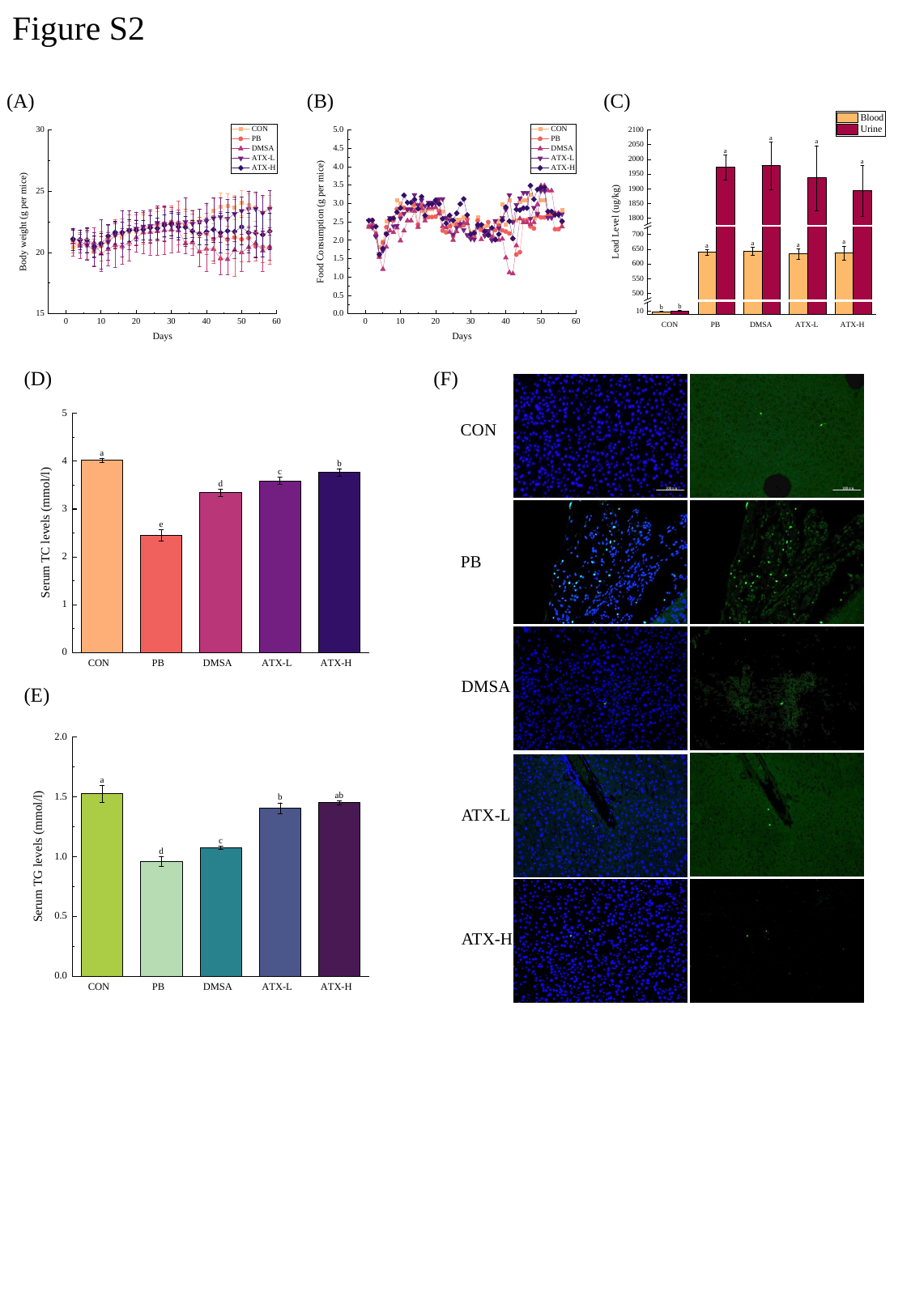

Figure S2
(A)
(C)
(B)
(F)
(D)
CON
PB
DMSA
ATX-L
ATX-H
(E)

## Slide 3
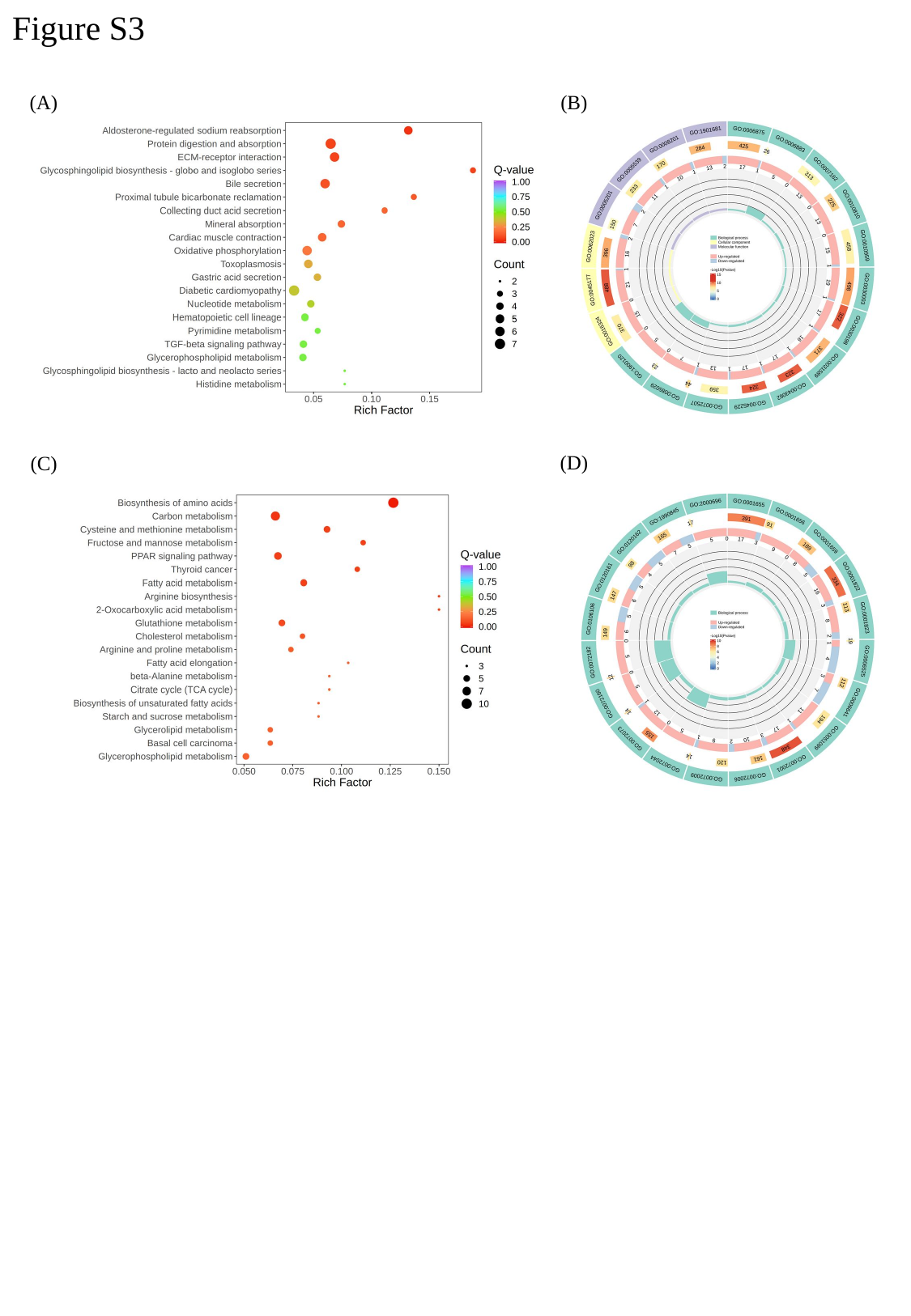

Figure S3
(A)
(B)
(D)
(C)
