# Supplementary material for: Astaxanthin Alleviates Lead‐Induced Toxicity by Restoring Hepatic and Gut–Liver Axis Homeostasis Through Multidimensional Metabolic and Antioxidative Pathways
Source: Food Sci Nutr. 2025 Sep 26;13(10):e70971. doi: 10.1002/fsn3.70971 (PMC12464569; doi:10.1002/fsn3.70971)
Supplement: Supplementary file 2 — Table S1: Composition of experimental diet. [file FSN3-13-e70971-s002.docx]

Table S1 The composition of experimental diet

| Ingredient | Per group/g |
| --- | --- |
| Sucrose | 331.77 |
| Cornstarch | 298.59 |
| casein | 189.58 |
| Cellulose | 47.4 |
| Maltodextrin | 33.18 |
| Soybean oil | 23.7 |
| Lard | 18.96 |
| Patassium citrate | 15.64 |
| Calcium hydrogenphosphate | 12.32 |
| M1002 mineral mix | 9.48 |
| V1001 vitamin mixture | 9.48 |
| Calcium carbonate | 5.21 |
| L-cystine | 2.84 |
| Choline bitartrate | 1.9 |
| Food dye | 0.047 |
| Total | 1000 |
