# Supplementary material for: Astaxanthin Alleviates Lead‐Induced Toxicity by Restoring Hepatic and Gut–Liver Axis Homeostasis Through Multidimensional Metabolic and Antioxidative Pathways
Source: Food Sci Nutr. 2025 Sep 26;13(10):e70971. doi: 10.1002/fsn3.70971 (PMC12464569; doi:10.1002/fsn3.70971)
Supplement: Supplementary file 3 — Table S2: Primers used for qRT–PCR. [file FSN3-13-e70971-s003.docx]

Table S2 Primers used for qRT–PCR

| Gene | Primer |
| --- | --- |
| *GADPH* | F: CGTGCCGCCTGGAGAAACCTG |
|  | R: AGAGTGGGAGTTGCTGTTGAAGTCG |
| Sult5a1 | F: GACTCTGGACCTTCTATGTG |
|  | R: GATGACCTGTGGCTTCTG |
| Cyp17a1 | F: AAGTGCTCGTGAAGAAGG |
|  | R: CCGTCGTATGTAAGTATCAAG |
| Aqp1 | F: CTCCAGGCACAGTCTTCT |
|  | R: GGCTCTCGGTTCACAATG |
| Igf1r | F: ATTCTGATGTCTGGTCCTTC |
|  | R: TGTTCTCCTCGCTGTAGTA |
| Ggt1 | F: CCACCTTCATCGCTGTAG |
|  | R: CCACTCTTGCTCCTGTTC |
| Gpx3 | F: GAGACCAGCCAAGACAAC |
|  | R: ACAGAGTGAGAGGATAGCAT |
| Slc22a22 | F: CCTTCCACATACTGACATTATC |
|  | R: AACGGATTGGCTAAGAGATT |
| Fxyd2 | F: GACTCTCGCTAACTTATCCTA |
|  | R: CATCTGCTATTGTGCTTACC |
