# Supplementary material for: Astaxanthin Alleviates Lead‐Induced Toxicity by Restoring Hepatic and Gut–Liver Axis Homeostasis Through Multidimensional Metabolic and Antioxidative Pathways
Source: Food Sci Nutr. 2025 Sep 26;13(10):e70971. doi: 10.1002/fsn3.70971 (PMC12464569; doi:10.1002/fsn3.70971)
Supplement: Supplementary file 4 — Table S3: Quality evaluation of mouse liver transcriptome sequencing data. [file FSN3-13-e70971-s008.docx]

Table S3 Quality evaluation of mouse liver transcriptome sequencing data

| Sample | Raw Reads | Clean Reads | Clean Base(G) | Error Rate(%) | Q20(%) | Q30(%) | GC Content(%) |
| --- | --- | --- | --- | --- | --- | --- | --- |
| ATX-H-1 | 46055764 | 45477670 | 6.82 | 0.03 | 97.85 | 93.52 | 39.29 |
| ATX-H-2 | 47859316 | 46919766 | 7.04 | 0.03 | 97.4 | 92.69 | 41.43 |
| ATX-H-3 | 54399004 | 53247482 | 7.99 | 0.03 | 97.57 | 92.97 | 40.96 |
| ATX-L-1 | 46604592 | 45821068 | 6.87 | 0.03 | 97.51 | 92.8 | 40.41 |
| ATX-L-2 | 44523282 | 43758112 | 6.56 | 0.03 | 97.58 | 92.89 | 39.84 |
| ATX-L-3 | 45627118 | 45058880 | 6.76 | 0.03 | 97.64 | 93.03 | 39.24 |
| CON-1 | 55947100 | 54732126 | 8.21 | 0.03 | 97.76 | 93.28 | 39.79 |
| CON-2 | 55458138 | 54675424 | 8.2 | 0.03 | 97.81 | 93.34 | 38.87 |
| CON-3 | 60168820 | 59120262 | 8.87 | 0.03 | 97.8 | 93.43 | 40.09 |
| DMSA-1 | 49634888 | 46591992 | 6.99 | 0.03 | 97.71 | 93.34 | 41.46 |
| DMSA-2 | 54080878 | 53090030 | 7.96 | 0.03 | 97.77 | 93.45 | 42.54 |
| DMSA-3 | 43438670 | 42423464 | 6.36 | 0.03 | 97.19 | 92.31 | 44.08 |
| Pb-1 | 57259112 | 56110296 | 8.42 | 0.03 | 97.35 | 92.63 | 42.27 |
| Pb-2 | 65305998 | 64242896 | 9.64 | 0.03 | 97.74 | 93.29 | 41.13 |
| Pb-3 | 60673774 | 59707014 | 8.96 | 0.03 | 97.6 | 92.98 | 39.38 |
