# Supplementary material for: Astaxanthin Alleviates Lead‐Induced Toxicity by Restoring Hepatic and Gut–Liver Axis Homeostasis Through Multidimensional Metabolic and Antioxidative Pathways
Source: Food Sci Nutr. 2025 Sep 26;13(10):e70971. doi: 10.1002/fsn3.70971 (PMC12464569; doi:10.1002/fsn3.70971)
Supplement: Supplementary file 5 — Table. S4 Identification of potential metabolite biomarkers both in CON vs. Pb and Pb vs. ATX‐H. [file FSN3-13-e70971-s007.docx]

Table S4 Identification of potential metabolite biomarkers both in CON vs Pb and Pb vs AST-H based on the criteria of a FC ≥ 2or≤0.5 and VIP ≥ 1

| Compounds | Class | CON vs Pb | | |  | Pb vs AST-H | | | Ko names of related pathway |
| --- | --- | --- | --- | --- | --- | --- | --- | --- | --- |
|  |  | VIP | FC | Trend |  | VIP | FC | Trend |  |
| 1,2-epoxy-3-(p-nitrophenoxy) propane | Benzene and substituted derivatives | 1.27 | 2.69 | up |  | 1.39 | 0.42 | down | - |
| 1,5-Diaminopentane | Alcohol and amines | 1.94 | 0.00 | down |  | 2.27 | 3087.48 | up | ko00310,ko00470,ko00480,ko01100,ko04974 |
| 1-Methylguanosine | Nucleotide And Its metabolites | 1.30 | 2.15 | up |  | 1.67 | 0.41 | down | - |
| 1-Methylhistidine | Amino acid and Its metabolites | 1.25 | 2.74 | up |  | 1.28 | 0.42 | down | ko00340,ko01100 |
| 1-Methylinosine | Nucleotide And Its metabolites | 1.59 | 3.34 | up |  | 1.71 | 0.38 | down | - |
| 11β-Prostaglandin E2 | FA | 1.63 | 13.37 | up |  | 1.29 | 0.24 | down | - |
| 2-(Dimethylamino)Guanosine | Nucleotide And Its metabolites | 1.15 | 2.62 | up |  | 1.62 | 0.29 | down | - |
| 2-Methylguanosine | Nucleotide And Its metabolites | 1.29 | 2.37 | up |  | 1.53 | 0.42 | down | - |
| 3-Indoleacrylic acid | Organic acid And Its derivatives | 1.78 | 0.43 | down |  | 1.57 | 2.00 | up | - |
| 4-Acetylaminobenzoic acid | Organic acid And Its derivatives | 1.58 | 3.65 | up |  | 1.75 | 0.32 | down | - |
| 5-Aminovaleric Acid | Amino acid and Its metabolites | 1.12 | 2.60 | up |  | 1.37 | 0.37 | down | ko00310,ko00330,ko00470,ko01100 |
| 6-keto-PGF1α | FA | 1.69 | 28.09 | up |  | 1.43 | 0.16 | down | ko00590,ko01100 |
| 7-Methylguanosine | Nucleotide And Its metabolites | 1.30 | 2.15 | up |  | 1.67 | 0.41 | down | - |
| Acetaminophen | Benzene and substituted derivatives | 1.13 | 2.05 | up |  | 1.35 | 0.44 | down | ko04976 |
| Carnitine C20:1-OH | FA | 1.67 | 3.46 | up |  | 1.89 | 0.32 | down | - |
| Carnitine C2:0 | FA | 1.05 | 4.83 | up |  | 1.35 | 0.18 | down | ko04931 |
| Carnitine C3:0 | FA | 1.66 | 2.30 | up |  | 1.82 | 0.49 | down | - |
| Carnitine C4:0 | FA | 1.60 | 7.06 | up |  | 1.39 | 0.32 | down | - |
| Carnitine isoC4:0 | FA | 1.60 | 7.06 | up |  | 1.39 | 0.32 | down | - |
| Cortisol | Hormones and hormone related compounds | 1.34 | 3.83 | up |  | 1.73 | 2.94 | up | ko00140,ko01100,ko04080,ko04927,ko04934,ko04960,ko04976,ko05200,ko05215 |
| D-(+)-sucrose | Carbohydrates and Its metabolites | 1.12 | 0.38 | down |  | 1.32 | 2.56 | up | ko00052,ko00500,ko01100,ko02010,ko04742,ko04973 |
| Glu-Met | Amino acid and Its metabolites | 1.39 | 5.63 | up |  | 1.11 | 0.32 | down | - |
| Glutathione Reducedform | Amino acid and Its metabolites | 1.05 | 0.38 | down |  | 1.16 | 2.21 | up | ko00270,ko00480,ko01100,ko01240,ko02010,ko04216,ko04918,ko04976,ko05208,ko05415 |
| Guanidineacetic Acid | Organic acid And Its derivatives | 1.12 | 3.92 | up |  | 1.37 | 0.24 | down | ko00260,ko00330,ko01100 |
| Hippuric Acid | Organic acid And Its derivatives | 1.58 | 3.65 | up |  | 1.75 | 0.32 | down | ko01100 |
| L-Arginine | Amino acid and Its metabolites | 1.28 | 76.47 | up |  | 1.35 | 0.02 | down | ko00220,ko00330,ko00470,ko00970,ko01100,ko01230,ko02010,ko04150,ko04974,ko05014,ko05022,ko05142,ko05146,ko05230 |
| L-threo-3-Methylaspartate | Amino acid and Its metabolites | 1.45 | 3.70 | up |  | 1.47 | 0.35 | down | ko00630,ko01100,ko01200 |
| Met-Glu | Amino acid and Its metabolites | 1.32 | 4.37 | up |  | 1.04 | 0.37 | down | - |
| N-Acetylaspartate | Amino acid and Its metabolites | 1.79 | 0.07 | down |  | 1.65 | 2.84 | up | ko00250,ko01100 |
| N-Amidino-L-Aspartate | Amino acid and Its metabolites | 1.93 | 0.08 | down |  | 2.07 | 2.02 | up | - |
| N-Methyl-D-Aspartic Acid | Amino acid and Its metabolites | 1.33 | 4.71 | up |  | 1.77 | 0.15 | down | - |
| N-acetylornithine | Amino acid and Its metabolites | 1.88 | 159265.01 | up |  | 1.10 | 0.05 | down | ko00220,ko01100,ko01210,ko01230 |
| N-glutarylglycine | Amino acid and Its metabolites | 1.15 | 0.47 | down |  | 1.42 | 2.48 | up | - |
| N-heptanoylglycine | Amino acid and Its metabolites | 1.74 | 0.34 | down |  | 1.56 | 2.36 | up | - |
| N6-(2-Hydroxyethyl)adenosine | Nucleotide And Its metabolites | 1.16 | 2.24 | up |  | 1.51 | 0.40 | down | - |
| O-Acetyl-L-serine | Amino acid and Its metabolites | 1.33 | 4.71 | up |  | 1.77 | 0.15 | down | ko00270,ko00920,ko01100,ko01200,ko01230,ko04122 |
| PC(O-16:0/0:0) | GP | 1.25 | 6.72 | up |  | 1.06 | 0.25 | down | - |
| Phosphoenolpyruvate | Organic acid And Its derivatives | 1.09 | 2.26 | up |  | 1.10 | 0.49 | down | ko00010,ko00020,ko00400,ko00440,ko00620,ko01100,ko01200,ko01230,ko01240,ko04922,ko04964,ko05230 |
| Prostaglandin E2 | FA | 1.63 | 13.37 | up |  | 1.29 | 0.24 | down | ko00590,ko01100,ko04024,ko04080,ko04625,ko04726,ko04750,ko04921,ko04923,ko04924,ko04976,ko05140,ko05143,ko05146,ko05163,ko05165,ko05200,ko05323 |
| Taurochenodesoxycholic Acid | Bile acids | 1.23 | 0.37 | down |  | 1.28 | 2.13 | up | ko00120,ko04976,ko04979 |
| Tricarballylic acid | Organic acid And Its derivatives | 1.86 | 16.45 | up |  | 1.97 | 0.21 | down | - |
| Uric acid | Organic acid And Its derivatives | 1.22 | 3.29 | up |  | 1.43 | 0.30 | down | ko00230,ko01100,ko04976 |
| Uridine 5’-Diphosphate | Nucleotide And Its metabolites | 1.04 | 0.46 | down |  | 1.17 | 2.10 | up | ko00240,ko01100,ko01232,ko01240,ko04080 |
| Uridine-5'-diphospho-N-acetylgalactosamine disodium salt | Nucleotide And Its metabolites | 1.16 | 0.42 | down |  | 1.40 | 2.28 | up | ko00520,ko00524,ko01100,ko01250,ko04931,ko05415 |
| estrone 3-sulfate | Hormones and hormone related compounds | 1.30 | 0.41 | down |  | 1.39 | 2.75 | up | ko00140,ko01100,ko04976 |
| p-Tolyl Sulfate | Organic acid And Its derivatives | 1.89 | 8.61 | up |  | 1.84 | 0.35 | down | - |
