# Supplementary material for: Astaxanthin Alleviates Lead‐Induced Toxicity by Restoring Hepatic and Gut–Liver Axis Homeostasis Through Multidimensional Metabolic and Antioxidative Pathways
Source: Food Sci Nutr. 2025 Sep 26;13(10):e70971. doi: 10.1002/fsn3.70971 (PMC12464569; doi:10.1002/fsn3.70971)
Supplement: Supplementary file 6 — Table. S5 Identification of potential exclusive metabolite biomarkers in CON vs. Pb. [file FSN3-13-e70971-s004.docx]

Table S5 Identification of potential exclusive metabolite biomarkers in CON vs Pb based on the criteria of a FC ≥ 2or≤0.5 and VIP ≥ 1

| Compounds | Class | CON vs Pb | | | Ko names of related pathway |
| --- | --- | --- | --- | --- | --- |
|  |  | VIP | FC | Trend |  |
| (+/-)-High-Proline | Amino acid and Its metabolites | 1.28 | 0.48 | down | - |
| (R)-(-)-Mandelic acid | Organic acid And Its derivatives | 1.70 | 2.76 | up | - |
| (±)12-HETE | FA | 1.43 | 2.70 | up | - |
| (±)15-HETE | FA | 1.51 | 2.44 | up | ko00590,ko04750 |
| (±)17-HDHA | FA | 1.56 | 3.35 | up | - |
| 1-Methyladenosine | Nucleotide And Its metabolites | 1.21 | 4.22 | up | - |
| 1-Methylxanthine | Nucleotide And Its metabolites | 1.03 | 7.93 | up | ko00232,ko01100 |
| 10-Formyl-7,8-dihydrofolic acid | Heterocyclic compounds | 1.74 | 2.01 | up | - |
| 11,12-EET | FA | 1.61 | 3.62 | up | - |
| 11-Dehydrocorticosterone | Hormones and hormone related compounds | 1.15 | 33.18 | up | ko00140 |
| 11-HDoHE | FA | 1.74 | 2.20 | up | - |
| 11-HEDE | FA | 1.61 | 2.33 | up | - |
| 12-HHT | FA | 1.72 | 5.68 | up | - |
| 13-HDoHE | FA | 1.51 | 2.07 | up | - |
| 13-HOTrE | FA | 1.04 | 2.08 | up | - |
| 14(S)-HDHA | FA | 1.66 | 4.37 | up | - |
| 15(R)-Prostaglandin E1 | FA | 1.79 | 3.70 | up | - |
| 15-HEDE | FA | 1.61 | 2.33 | up | - |
| 15-oxoETE | FA | 1.33 | 2.62 | up | ko00590 |
| 16,16-Dimethyl PGA2 | FA | 1.50 | 3.84 | up | - |
| 16-Hydroxyhexadecanoic acid | FA | 1.94 | 2.82 | up | ko01100 |
| 2'-Aenylic Acid | Nucleotide And Its metabolites | 1.21 | 0.43 | down | - |
| 2'-Deoxycytidine-5'-Monophosphate | Nucleotide And Its metabolites | 1.13 | 0.42 | down | ko00240,ko01100,ko01232 |
| 2'-O-methyladenosine | Nucleotide And Its metabolites | 1.12 | 0.42 | down | - |
| 2,6-Diamino-5-hydroxyhexanoic acid | Amino acid and Its metabolites | 1.10 | 3.99 | up | ko00310,ko01100 |
| 2-Methylsuccinic Acid | Organic acid And Its derivatives | 1.82 | 2.56 | up | - |
| 2-hydroxyhexadecanoic acid | FA | 1.94 | 2.82 | up | - |
| 2-keto-D-gluconic acid | Carbohydrates and Its metabolites | 1.10 | 4.59 | up | ko00030,ko01100 |
| 2-methyl citric acid | Organic acid And Its derivatives | 1.82 | 2.23 | up | - |
| 21-Deoxycortisol | Hormones and hormone related compounds | 1.32 | 22.66 | up | ko00140 |
| 3'-Adenylic acid | Nucleotide And Its metabolites | 1.21 | 0.43 | down | ko00230,ko01100 |
| 3'-Dephosphocoenzyme A | CoEnzyme and vitamins | 1.44 | 2.01 | up | ko00770,ko01100,ko01240 |
| 3-Deoxyguanosine | Nucleotide And Its metabolites | 1.40 | 2.33 | up | - |
| 3-Hydroxypicolinic acid | Heterocyclic compounds | 1.28 | 0.47 | down | - |
| 3-Indolepropionic Acid | Heterocyclic compounds | 1.67 | 2.36 | up | - |
| 6-aminonicotinamide | Nucleotide And Its metabolites | 1.77 | 0.38 | down | - |
| 8-iso Prostaglandin E1 | FA | 1.88 | 3.97 | up | - |
| Adenosine 5'-Monophosphate | Nucleotide And Its metabolites | 1.14 | 0.43 | down | ko00230,ko01100,ko01232,ko01240,ko01523,ko04022,ko04024,ko04068,ko04150,ko04151,ko04152,ko04211,ko04740,ko04742,ko04923,ko04924,ko04925,ko04927,ko04928,ko04934,ko05012,ko05022,ko05032 |
| Arg-Val | Amino acid and Its metabolites | 1.36 | 3.37 | up | - |
| Biotinamide | Alcohol and amines | 1.77 | 3.25 | up | - |
| CCT-018159 | Benzene and substituted derivatives | 1.55 | 0.24 | down | - |
| Carnitine C13:1 | FA | 1.54 | 0.16 | down | - |
| Carnitine C14:0 | FA | 1.49 | 4.93 | up | - |
| Carnitine C16-OH | FA | 1.76 | 2.11 | up | - |
| Carnitine C18:1 | FA | 1.65 | 2.55 | up | - |
| Carnitine C18:1-OH | FA | 1.66 | 2.02 | up | - |
| Carnitine C5:0 | FA | 1.12 | 2.53 | up | - |
| Carnitine C5:1 | FA | 1.71 | 3.03 | up | - |
| Carnitine C6:0 | FA | 1.38 | 2.07 | up | - |
| Carnitine C8-OH | FA | 1.80 | 2.45 | up | - |
| Carnitine ph-C1 | FA | 1.90 | 3.39 | up | - |
| Carnitine-2-methyl-C4 | FA | 1.12 | 2.53 | up | - |
| Cholic acid | Bile acids | 1.31 | 0.19 | down | ko00120,ko01100,ko04976 |
| Citraconic Acid | Organic acid And Its derivatives | 1.06 | 4.73 | up | ko00290,ko01100,ko01210 |
| Cortexolone | Hormones and hormone related compounds | 1.47 | 67.22 | up | ko00140,ko01100,ko04927,ko04934 |
| Corticosterone | Hormones and hormone related compounds | 1.61 | 87.10 | up | ko00140,ko01100,ko04923,ko04925,ko05020 |
| Cyclamic acid | Organic acid And Its derivatives | 1.67 | 2.04 | up | - |
| Cys-Gly | Amino acid and Its metabolites | 1.20 | 0.26 | down | ko00480,ko01100 |
| Cytidine 2',3'-cyclic monophosphoric acid | Nucleotide And Its metabolites | 1.16 | 2.70 | up | ko00240,ko01100 |
| D-Galacturonic Acid | Carbohydrates and Its metabolites | 1.06 | 4.30 | up | ko00040,ko00053,ko00520,ko01100,ko01240,ko01250,ko02010 |
| D-Glucoronic Acid | Carbohydrates and Its metabolites | 1.04 | 4.87 | up | - |
| D-Maltopentaose | Carbohydrates and Its metabolites | 1.08 | 0.24 | down | - |
| D-Trehalose | Carbohydrates and Its metabolites | 1.39 | 0.36 | down | ko00500,ko01100,ko02010 |
| DL-2-hydroxystearic acid | FA | 1.91 | 2.17 | up | - |
| Deoxycholic acid | Bile acids | 1.13 | 0.44 | down | ko04976 |
| Deoxyguanosine 5'-monophosphate(dGMP) | Nucleotide And Its metabolites | 1.13 | 0.46 | down | ko00230,ko01100,ko01232 |
| Dextrin | Carbohydrates and Its metabolites | 1.12 | 0.32 | down | ko00500,ko01100 |
| Dimethylmalonic acid | Organic acid And Its derivatives | 1.82 | 2.56 | up | - |
| EPA | FA | 1.37 | 2.40 | up | ko01040 |
| Ethyl hydrogen malonate | Aldehyde,Ketones,Esters | 1.82 | 2.56 | up | - |
| Ethylmalonate | Organic acid And Its derivatives | 1.83 | 2.42 | up | - |
| FFA(18:4) | FA | 1.77 | 0.32 | down | ko00592 |
| FFA(22:7) | FA | 1.73 | 3.16 | up | - |
| Fumaric Acid | Organic acid And Its derivatives | 1.01 | 2.23 | up | ko00020,ko00190,ko00220,ko00250,ko00350,ko00360,ko00620,ko00650,ko00760,ko01100,ko01200,ko04922,ko05200,ko05211,ko05230 |
| GalactinolHydrate | Carbohydrates and Its metabolites | 1.11 | 0.44 | down | ko00052,ko01100 |
| Gamma-Mercholic Acid | Bile acids | 1.31 | 0.19 | down | - |
| Glu-Leu | Amino acid and Its metabolites | 1.12 | 2.53 | up | - |
| Glu-Tyr | Amino acid and Its metabolites | 1.18 | 2.04 | up | - |
| Gluceptate | Carbohydrates and Its metabolites | 1.65 | 2.24 | up | - |
| Glutaconic acid | Amino acid and Its metabolites | 1.06 | 4.73 | up | - |
| Glutaric Acid | Organic acid And Its derivatives | 1.82 | 2.56 | up | ko00071,ko00310,ko01100 |
| Glycocholic Acid | Bile acids | 1.21 | 0.43 | down | ko00120,ko01100,ko04976,ko04979 |
| Glycogen | Carbohydrates and Its metabolites | 1.20 | 0.28 | down | ko04922 |
| Glycohyodeoxycholic acid | Bile acids | 1.19 | 0.47 | down | - |
| His-Tyr | Amino acid and Its metabolites | 1.22 | 0.50 | down | - |
| Homovanillic Acid sulfate (sodium salt) | Organic acid And Its derivatives | 1.72 | 2.00 | up | - |
| Hyodeoxycholic acid | Bile acids | 1.30 | 0.22 | down | - |
| Hypoxanthine | Nucleotide And Its metabolites | 1.43 | 2.39 | up | ko00230,ko01100,ko01232 |
| Iminodiacetic acid | Organic acid And Its derivatives | 1.58 | 2.74 | up | - |
| Indoxylsulfuric acid | Heterocyclic compounds | 1.83 | 2.84 | up | - |
| Isocitric acid | Organic acid And Its derivatives | 1.30 | 66.49 | up | ko00020,ko00630,ko01100,ko01200,ko01210,ko01230,ko01240,ko04922,ko05230 |
| Isomaltotriose | Carbohydrates and Its metabolites | 1.12 | 0.32 | down | ko02010 |
| Kojibiose | Others | 1.11 | 0.33 | down | - |
| L-Thyroxine | Hormones and hormone related compounds | 1.51 | 0.46 | down | ko00350,ko01100,ko04080,ko04918,ko04919,ko04976,ko05320 |
| L-rhamnonic acid | Carbohydrates and Its metabolites | 1.52 | 2.22 | up | ko00051,ko01100 |
| LPC(O-16:1/0:0) | GP | 1.22 | 4.40 | up | - |
| LPC(O-18:1/0:0) | GP | 1.56 | 2.61 | up | - |
| LPE(0:0/18:2) | GP | 1.52 | 0.47 | down | - |
| LPE(0:0/18:3) | GP | 1.91 | 0.23 | down | - |
| LPE(18:3/0:0) | GP | 1.91 | 0.23 | down | - |
| Lactose | Carbohydrates and Its metabolites | 1.39 | 0.36 | down | ko00052,ko01100,ko02010,ko04973 |
| Lactulose | Carbohydrates and Its metabolites | 1.39 | 0.36 | down | - |
| Lys-Tyr | Amino acid and Its metabolites | 1.18 | 0.37 | down | - |
| Maltose | Carbohydrates and Its metabolites | 1.39 | 0.36 | down | ko00500,ko01100,ko02010,ko04742,ko04973 |
| N-Acetyl-L-Histidine | Amino acid and Its metabolites | 1.07 | 0.46 | down | - |
| N-Acetyl-L-phenylalanine | Amino acid and Its metabolites | 1.30 | 0.45 | down | ko00360,ko01100 |
| N-Arachidonic-acid-L-serine | FA | 1.14 | 2.08 | up | - |
| N-Methylisoleucine | Amino acid and Its metabolites | 1.81 | 2.07 | up | - |
| N-methyl-4-aminobutyric acid | Amino acid and Its metabolites | 1.18 | 0.47 | down | - |
| N-γ-Acetyl-N-2-Formyl-5-Methoxykynurenamine | Amino acid and Its metabolites | 1.41 | 0.42 | down | ko00380 |
| N6-methyladenosine | Nucleotide And Its metabolites | 1.21 | 4.22 | up | - |
| O-Phosphorylethanolamine | GP | 1.68 | 3.88 | up | ko00563,ko00564,ko00600,ko01100,ko04071 |
| Octadecanedioic acid | Organic acid And Its derivatives | 1.54 | 0.49 | down | - |
| PGD1 | FA | 1.54 | 2.76 | up | - |
| PGD2 | FA | 1.68 | 2.33 | up | ko00590,ko01100,ko04080,ko04664,ko04726,ko05143,ko05310 |
| PGE1 | FA | 1.54 | 2.76 | up | - |
| PGF1α | FA | 1.77 | 3.21 | up | - |
| PGJ2 | FA | 1.77 | 2.86 | up | ko00590,ko01100,ko04726 |
| Palatinose | Heterocyclic compounds | 1.35 | 0.46 | down | - |
| Phe-His | Amino acid and Its metabolites | 1.25 | 0.39 | down | - |
| Phosphatidylethanolamine lyso alkenyl 16:0 | GP | 1.46 | 2.04 | up | - |
| Phosphatidylethanolamine lyso alkenyl 18:2 | GP | 1.46 | 12.18 | up | - |
| Pregnanetriol | Alcohol and amines | 1.57 | 0.49 | down | - |
| Prostaglandin F2α | FA | 1.85 | 5.42 | up | ko00590,ko01100,ko04072,ko04080,ko04726,ko04913,ko04921,ko04976 |
| S-(Methyl)glutathione | Amino acid and Its metabolites | 1.07 | 0.43 | down | - |
| S-Allyl-L-cysteine | Amino acid and Its metabolites | 1.60 | 3.28 | up | - |
| S-nitrosoglutathione | Amino acid and Its metabolites | 1.27 | 0.35 | down | - |
| ST-638 | Organic acid And Its derivatives | 1.59 | 0.14 | down | - |
| Sebacate | Organic acid And Its derivatives | 1.24 | 2.13 | up | - |
| Sphingosyl-phosphocholine | SL | 1.40 | 4.42 | up | - |
| Stachyose | Carbohydrates and Its metabolites | 1.28 | 0.32 | down | ko00052,ko01100 |
| TXB2 | FA | 1.84 | 4.25 | up | ko00590,ko01100,ko04726,ko04976 |
| Tauro-beta-muricholic acid | Bile acids | 1.42 | 0.23 | down | - |
| Taurohyocholic acid | Bile acids | 1.42 | 0.23 | down | - |
| Tauroursodeoxycholic acid | Bile acids | 1.44 | 0.24 | down | - |
| Thiamine | CoEnzyme and vitamins | 1.74 | 0.46 | down | ko00730,ko01100,ko01240,ko02010,ko04122,ko04977 |
| Thiamine-Monophosphate | Heterocyclic compounds | 1.93 | 0.39 | down | ko00730,ko01100,ko01240 |
| Thymine | Nucleotide And Its metabolites | 1.61 | 3.90 | up | ko00240,ko01100,ko01232 |
| Trp-Gly | Amino acid and Its metabolites | 1.18 | 2.75 | up | - |
| Tyr-His | Amino acid and Its metabolites | 1.43 | 0.42 | down | - |
| URB937 | Benzene and substituted derivatives | 1.40 | 0.15 | down | - |
| alpha-Muricholic acid | Bile acids | 1.31 | 0.19 | down | - |
| beta-Muricholic acid | Bile acids | 1.31 | 0.19 | down | - |
| ent-Prostaglandin F2α | FA | 1.88 | 3.97 | up | - |
| gamma-Glu-Ala | Amino acid and Its metabolites | 1.35 | 2.11 | up | ko00480,ko01100 |
| γ-Glu-Met | Amino acid and Its metabolites | 1.20 | 4.29 | up | - |
