# Supplementary material for: Astaxanthin Alleviates Lead‐Induced Toxicity by Restoring Hepatic and Gut–Liver Axis Homeostasis Through Multidimensional Metabolic and Antioxidative Pathways
Source: Food Sci Nutr. 2025 Sep 26;13(10):e70971. doi: 10.1002/fsn3.70971 (PMC12464569; doi:10.1002/fsn3.70971)
Supplement: Supplementary file 7 — Table. S6 Identification of potential exclusive metabolite biomarkers in Pb vs. ATX‐H. [file FSN3-13-e70971-s005.docx]

Table S6 Identification of potential exclusive metabolite biomarkers in Pb vs AST-H based on the criteria of a FC ≥ 2or≤0.5 and VIP ≥ 1

| Compounds | Class | Pb vs AST-H | | | Ko names of related pathway |
| --- | --- | --- | --- | --- | --- |
|  |  | VIP | FC | Trend |  |
| (E)-3-(3-hydroxy-4-methoxyphenyl)acrylic acid | Benzene and substituted derivatives | 1.00 | 2.30 | up | - |
| (E,Z)-2-Amino-3,14-octadecadien-1-ol | Alcohol and amines | 1.22 | 0.36 | down | - |
| (±)4-HDHA | FA | 1.03 | 0.35 | down | - |
| 10-Hydroxystearic Acid | FA | 1.14 | 8.63 | up | - |
| 11-Cis-Retinol | CoEnzyme and vitamins | 1.11 | 2.14 | up | ko00830,ko01100 |
| 12,13-DiHOME | FA | 1.51 | 2.85 | up | - |
| 2-Methylhippuric acid | Amino acid and Its metabolites | 1.74 | 0.44 | down | ko01100 |
| 2-hydroxyphenylacetic acid | Organic acid And Its derivatives | 2.00 | 2.39 | up | ko00360,ko01100 |
| 3-Hydroxyphenylacetic acid | Organic acid And Its derivatives | 1.71 | 3.54 | up | ko00350,ko00360,ko01100 |
| 3-N-Methyl-L-Histidine | Amino acid and Its metabolites | 1.11 | 2.26 | up | ko00340,ko01100 |
| 4-Hydroxy-3-methylbenzoic acid | Organic acid And Its derivatives | 1.88 | 2.25 | up | - |
| 5-Hydroxyindole-3-Acetic Acid | Heterocyclic compounds | 1.10 | 0.41 | down | ko00380,ko01100,ko04726 |
| 9(S)-HOTrE | FA | 1.37 | 2.10 | up | ko00592 |
| 9,10-DiHOME | FA | 1.15 | 2.69 | up | ko00591 |
| Adenylocuccinic Acid | Organic acid And Its derivatives | 1.43 | 2.79 | up | ko00230,ko00250,ko01100,ko01232,ko01240 |
| All-Trans-13,14-Dihydroretinol | CoEnzyme and vitamins | 1.13 | 3.64 | up | ko00830 |
| D-Calcium Pantothenate | CoEnzyme and vitamins | 1.29 | 0.24 | down | - |
| Dehydroabietic acid | Organic acid And Its derivatives | 1.62 | 2.65 | up | - |
| Ferulic acid | Organic acid And Its derivatives | 1.02 | 2.54 | up | ko01100 |
| Isoxanthopterin | Heterocyclic compounds | 1.25 | 2.05 | up | - |
| L-2-amino-6-oximelic acid | Organic acid And Its derivatives | 1.35 | 2.39 | up | ko01100,ko01230 |
| LPE(14:0/0:0) | GP | 1.76 | 2.42 | up | - |
| Mandelic Acid | Organic acid And Its derivatives | 1.82 | 3.54 | up | - |
| N-Acetyl-Asp-Glu | Amino acid and Its metabolites | 1.28 | 3.11 | up | ko00250,ko01100,ko04080 |
| N-Acetyl-L-Glutamic Acid | Amino acid and Its metabolites | 1.30 | 2.18 | up | ko00220,ko01100,ko01210,ko01230 |
| N-Butyrylglycine | Amino acid and Its metabolites | 1.98 | 2.83 | up | - |
| N-Cinnamylglycine | Organic acid And Its derivatives | 1.46 | 0.24 | down | - |
| N-Phenylacetylglycine | Amino acid and Its metabolites | 1.74 | 0.44 | down | ko00360 |
| Octadecadienamide | Alcohol and amines | 1.04 | 0.30 | down | - |
| Oleamide | Alcohol and amines | 1.22 | 0.36 | down | - |
| Phenylacetyl-L-Glutamine | Amino acid and Its metabolites | 1.72 | 0.43 | down | ko00360 |
| P–Hydroxyphenyl Acetic Acid | Benzene and substituted derivatives | 1.82 | 3.54 | up | ko00350,ko00360,ko01100 |
| Sphingosine 1-phosphate | SL | 1.76 | 2.89 | up | ko00600,ko01100,ko04020,ko04071,ko04072,ko04080,ko04371,ko04666,ko05152 |
| Tetradecanedioic acid | FA | 1.73 | 2.22 | up | - |
| Trans-3-Hydroxycotinine | Heterocyclic compounds | 1.17 | 2.48 | up | - |
| Triethanolamine | Alcohol and amines | 1.56 | 2.10 | up | ko00564 |
| Ureidoisobutyric Acid | Organic acid And Its derivatives | 1.31 | 2.19 | up | - |
| Β-Nicotinamide Mononucleotide | Nucleotide And Its metabolites | 1.22 | 0.28 | down | ko00760,ko01100 |
