# Supplementary material for: Astaxanthin Alleviates Lead‐Induced Toxicity by Restoring Hepatic and Gut–Liver Axis Homeostasis Through Multidimensional Metabolic and Antioxidative Pathways
Source: Food Sci Nutr. 2025 Sep 26;13(10):e70971. doi: 10.1002/fsn3.70971 (PMC12464569; doi:10.1002/fsn3.70971)
Supplement: Supplementary file 8 — Table S7: Annotation of metabolites involved in the heat map in Figure 8. [file FSN3-13-e70971-s001.docx]

Table S7 Annotation of metabolites involved in the heat map in Figure 8

| Index | Compounds | Class I | Class II |
| --- | --- | --- | --- |
| MEDN0758 | (±)4-HDHA | FA | Oxidized lipids |
| MEDP0408 | 11-Cis-Retinol | CoEnzyme and vitamins | CoEnzyme and vitamins |
| MEDN1009 | 3-Hydroxyphenylacetic acid | Organic acid And Its derivatives | Organic acid And Its derivatives |
| MEDP0272 | 5-Hydroxyindole-3-Acetic Acid | Heterocyclic compounds | Indole and Its derivatives |
| MEDN1416 | 9(S)-HOTrE | FA | Oxidized lipids |
| MEDP0244 | All-Trans-13,14-Dihydroretinol | CoEnzyme and vitamins | CoEnzyme and vitamins |
| MEDN1630 | Ferulic acid | Organic acid And Its derivatives | Organic acid And Its derivatives |
| MEDN2002 | N-Acetyl-L-Glutamic Acid | Amino acid and Its metabolites | Amino acid derivatives |
| MEDN1083 | Sphingosine 1-phosphate | SL | SM |
| MEDP0180 | Β-Nicotinamide Mononucleotide | Nucleotide And Its metabolites | Nucleotide And Its metabolites |
| MEDN0781 | 6-keto-PGF1α | FA | Oxidized lipids |
| MEDP1377 | Carnitine C20:1-OH | FA | CAR |
| MEDP0510 | Carnitine C2:0 | FA | CAR |
| MEDP1665 | Carnitine C3:0 | FA | CAR |
| MEDP1442 | Carnitine C4:0 | FA | CAR |
| MEDP0577 | Carnitine isoC4:0 | FA | CAR |
| MEDP0889 | Cortisol | Hormones and hormone related compounds | Hormones and hormone related compounds |
| MEDN0039 | Glutathione Reducedform | Amino acid and Its metabolites | Small Peptide |
| MEDN0317 | Hippuric Acid | Organic acid And Its derivatives | Organic acid And Its derivatives |
| MEDP0063 | N-Acetylaspartate | Amino acid and Its metabolites | Amino acid derivatives |
| MEDN1005 | Phosphoenolpyruvate | Organic acid And Its derivatives | Phosphoric acids |
| MEDN0793 | Prostaglandin E2 | FA | Oxidized lipids |
| MEDN1006 | Uric acid | Organic acid And Its derivatives | Organic acid And Its derivatives |
| MEDN0170 | Uridine 5’-Diphosphate | Nucleotide And Its metabolites | Nucleotide And Its metabolites |
| MEDN0536 | estrone 3-sulfate | Hormones and hormone related compounds | Hormones and hormone related compounds |
